# UCHL3 induces radiation resistance and acquisition of mesenchymal phenotypes by deubiquitinating POLD4 in glioma stem cells

**DOI:** 10.1007/s00018-024-05265-5

**Published:** 2024-06-03

**Authors:** Ligang Fan, Hongtao You, Xiao Jiang, Yixuan Niu, Zhengxin Chen, Huibo Wang, Yuan Xu, Peng Zhou, Li Wei, Tianwei Jiang, Danni Deng, Lian Xue, Ya Peng, Wei Xing, Naiyuan Shao

**Affiliations:** 1 Department of Neurosurgery, The Third Affiliated Hospital of Soochow University, Changzhou, 213003 Jiangsu Province China; 2 Clinical Medical Research Center, The Third Affiliated Hospital of Soochow University, Changzhou, 213003 Jiangsu Province China; 3 Department of Blood Transfusion, The Third Affiliated Hospital of Soochow University, Changzhou, 213003 Jiangsu Province China; 4 Department of Neurosurgery, First Affiliated Hospital of Nanjing Medical University, Nanjing, 210029 Jiangsu Province China; 5 Department of Radiology, Third Affiliated Hospital of Soochow University, Changzhou, China

**Keywords:** Glioma stem cells, Glioma stem cells, Irradiation resistance, UCHL3-POLD4 signaling

## Abstract

**Background:**

The high degree of intratumoral genomic heterogeneity is a major obstacle for glioblastoma (GBM) tumors, one of the most lethal human malignancies, and is thought to influence conventional therapeutic outcomes negatively. The proneural-to-mesenchymal transition (PMT) of glioma stem cells (GSCs) confers resistance to radiation therapy in glioblastoma patients. POLD4 is associated with cancer progression, while the mechanisms underlying PMT and tumor radiation resistance have remained elusive.

**Method:**

Expression and prognosis of the POLD family were analyzed in TCGA, the Chinese Glioma Genome Atlas (CGGA) and GEO datasets. Tumorsphere formation and in vitro limiting dilution assay were performed to investigate the effect of UCHL3-POLD4 on GSC self-renewal. Apoptosis, TUNEL, cell cycle phase distribution, modification of the Single Cell Gel Electrophoresis (Comet), γ-H2AX immunofluorescence, and colony formation assays were conducted to evaluate the influence of UCHL3-POLD4 on GSC in ionizing radiation. Coimmunoprecipitation and GST pull-down assays were performed to identify POLD4 protein interactors. In vivo, intracranial xenograft mouse models were used to investigate the molecular effect of UCHL3, POLD4 or TCID on GCS.

**Result:**

We determined that POLD4 was considerably upregulated in MES-GSCs and was associated with a meagre prognosis. Ubiquitin carboxyl terminal hydrolase L3 (UCHL3), a DUB enzyme in the UCH protease family, is a bona fide deubiquitinase of POLD4 in GSCs. UCHL3 interacted with, depolyubiquitinated, and stabilized POLD4. Both in vitro and in vivo assays indicated that targeted depletion of the UCHL3-POLD4 axis reduced GSC self-renewal and tumorigenic capacity and resistance to IR treatment by impairing homologous recombination (HR) and nonhomologous end joining (NHEJ). Additionally, we proved that the UCHL3 inhibitor TCID induced POLD4 degradation and can significantly enhance the therapeutic effect of IR in a gsc-derived in situ xenograft model.

**Conclusion:**

These findings reveal a new signaling axis for GSC PMT regulation and highlight UCHL3-POLD4 as a potential therapeutic target in GBM. TCID, targeted for reducing the deubiquitinase activity of UCHL3, exhibited significant synergy against MES GSCs in combination with radiation.

**Supplementary Information:**

The online version contains supplementary material available at 10.1007/s00018-024-05265-5.

## Introduction

Glioblastoma (GBM) is the most aggressive primary brain tumor in adults with a median survival of 15–20 months [1]. The standard of therapy for GBM includes maximal safe surgical resection followed by high-dose radiation therapy (RT) with concurrent and adjuvant temozolomide chemotherapy, yielding only a minor clinical benefit [2]. Functionally well-defined glioma stem cells (GSCs) contribute to this poor prognosis by driving therapeutic resistance and maintenance of cellular heterogeneity.

Similar to GBMs, gene expression analysis has discovered that GSCs can be classified into classical, mesenchymal (MES) or proneural (PN) subtypes; of these subtypes, PN tumors display the most relatively encouraging prognosis whereas MES subtypes present more impervious to radiotherapy and have the most aggressive phenotype [3, 4]. Numerous researchers and our team have reported that ionizing radiation (IR) to glioma spheres increases MES marker expression with a concomitant decrease in PN markers, raising the possibility that IR induces a PMT phenotypic shift in GSCs [5, 6]. Despite these advances about the central role of PMT in GBM recurrence, little is known about the precise molecular mechanisms responsible for the cellular phenotypic shift and behavioral plasticity to IR in GSCs.

Although IR damages tumor cells through several mechanisms, IR kills cancer cells primarily through DNA damage [7]. DNA double-strand breaks (DSBs), engendered through ionizing radiation (IR), are the most harmful DNA lesions, because the failure of DNA can lead to mutations and genomic rearrangements that could lead to enhanced tumorigenesis. However, these DNA lesions can induce cell death after a certain threshold [8]. DSBs are generally repaired by either error-free homologous recombination (HR) or error-prone nonhomologous end joining (NHEJ) pathways, depending on the context of the DNA damage [9–11]. In GBM, especially in GSCs, there is an enhanced DNA DSB repair, primarily HR, which has been linked to radio and chemoresistance in this type of tumor [12]. POLD4, namely, p12, the fourth smallest subunit of DNA replicative polymerase delta, is believed to contribute to the regulation of replication by facilitating repair in response to DNA damage [13]. Loss of POLD4 results in a defect in HR and sensitization to PARP inhibitors, indicating that an indispensable component of Polδ is necessary for functional HR [14]. POLD4 has been observed in the development and progression of multiple cancers, including hepatocellular carcinoma [15], lung cancer[16, 17], and cisplatin resistance in gastric cancer [18]. In GBMs, POLD4 is associated with cancer patient resistance to radiotherapy and tumor recurrence [19]. Nevertheless, the underlying specific mechanisms that enable POLD4 to promote GBM radiotherapy resistance remain unclear.

Ubiquitination is a widespread reversible posttranslational modification (PTM) that has emerged as a crucial mechanism that regulates protein maturation and plays critical roles in diverse biological processes in cancer initiation and progression [20]. This biological homoeostasis process is tightly regulated by deubiquitinating enzymes (DUBs). In a siRNA screening analysis that identified relevant genes for GBM survival, 22% were components of the 20S and 26S proteasome subunits [21]. In addition, several E2, E3, and DUB enzymes have been identified with protumorigenic or anticancer effects in GBM/GSCs [22]. UCHL3 is a significant member of the UCL subfamily that modulates numerous cellular processes, including cell viability and invasion [23, 24]. DNA damage repair and radioresistance [25–27], and stemness maintenance [28]. However, the function of UCHL3 in GBM radiation resistance and the role of UCHL3 in regulating GSC properties remain enigmatic.

In this study, we found that POLD4 expression correlates positively with MES GSC genes, negatively with PN GSC genes, and indicates poor survival in GBM, suggesting that POLD4 is enriched in the MES phenotype and that a higher level of POLD4 confers radiation resistance in GBM patients. Mechanistically, the deubiquitinase UCHL3 was shown to bind and stabilize POLD4, mediating the characteristics of GSCs, especially their self-renewal ability, radiotherapy resistance and tumorigenicity. We also evaluated the therapeutic efficiency of UCHL3 inhibition in combination with radiotherapy on patient-derived MES GSC xenografts.

## Materials and methods

### Human tissue samples

Samples were collected from 114 formalin-fixed, paraffin-embedded GBM patients who underwent surgical resection at the Department of Neurosurgery, the Third Affiliated Hospital of Soochow University, from 2009 to 2022. The histological sections of the samples were reviewed by two independent neuropathologists following the WHO standard classification guidelines. The study was approved by the Research Ethics Board of the Third Affiliated Hospital of Soochow University, and written consent was obtained from each participant.

### Cell lines, GSCs and cultures

Human embryonic kidney HEK293T cell lines were obtained from ATCC and cultivated in DMEM with 10% FBS. PN GSCs (PN35 and PN182) and MES GSCs (MES 21 and MES 505) were dissociated from primary GBM specimens or patient-derived GBM xenografts and functionally characterized as previously described [6, 29]. Cells were grown in serum-free DMEM/F12 medium, supplemented with B27 (1:50, Invitrogen), 20 ng/mL EGF and 20 ng/mL bFGF (BD Biosciences). Only GSCs from early passages (i.e., 3–8) were used for all studies herein. All cell lines were periodically tested for mycoplasma contamination using MycoAlert PLUS Kits from Lonza.

### Plasmid constructs and cotransfection

UCHL3, POLD4 and control RNA (shRNA) were obtained from Sigma–Aldrich and the targeting sequences are described in Supplemental Table 1. Flag-USP9X, Flag-USP22, Flag-UCHL1, Flag-UCHL3^WT^, Flag-UCHL3^S75A^, GST- UCHL3^WT^, GST-CHL3^S75A^, His-POLD4, HA-Ub, HA-Ub-K0, HA-Ub-K48, and HA-Ub-K63 overexpression plasmids were purchased from GeneChem. Recipient cells were transfected using Lipofectamine 3000 (Invitrogen, catalog L3000150) following the manufacturer’s instructions. Positive stable cell lines were selected by puromycin.

### orthotopic xenograft models

For all experiments, all mice were randomly allocated into groups. Stable GSCs (PN or MES) were obtained by expressing the plasmid pLenti-CMVPuro-LUC (Addgene) and selecting in the presence of 0.5 μg/ml puromycin. All constructs were transduced into different GSCs using lentiviral vectors. A total of 1 × 10^5^ GSCs expressing stable luciferase were implanted by stereotactic injection into the right striatum of 6–8-week-old nude mice. To assess the in vivo effect of UCHL3 inhibitor TCID, 1 × 10^5^ GSCs were implanted intracranially into individual mice. ALZET micro osmotic pumps (DURECT Corp.) and an infusion apparatus were implanted into tumour-bearing mice, and either TCID (10 mg/kg) or a vehicle was initiated. Treatment was delivered at a rate of 0.5 μl/h for 7 days. Tumor implantation was analyzed by bioluminescence imaging with an IVIS 200 Spectrum Imaging System on the indicated days to verify tumour growth during radiation. At approximately 60 days or in the presence of pathological signs after GSC implantation, the mice were humanely sacrificed, and their brains were collected, paraffin-embedded, stained with H&E to confirm the existence of tumours or subjected to immunohistochemical staining. For the animal survival analysis, mice were kept until the presence of pathological signs (i.e., ruffled coat, weight loss, back hunching, decreased appetite, and inability to move) from tumor burden developed or 60 days after injection. All animal experiments were approved by the Ethics Committee of the Third Affiliated Hospital of Soochow.

### Extreme limiting dilution analysis (ELDA)

Briefly, decreasing numbers of GSCs (with different constructs or treatment) per well (50, 20, 10, 5 and 1) were seeded into 96-well plates. Each well was examined for the formation of tumor spheres. Stem cell frequency was measured and analyzed by Extreme Limiting Dilution Analysis software (http://bioinf.wehi.edu.au/software/elda).

### γ-H2AX foci formation assay

GSCs transduced with different constructs or treatments were seeded into 16-well plates with poly-L-lysine-coated coverslips in a culture medium overnight at a cell number of 1◊10^4^ cells. Modified GSCs were then subjected to irradiation (6 Gy). The γ-H2AX foci were visualized by a DNA Damage Assay Kit by γ-H2AX immunofluorescence (Beyotime, C2035S) according to the manufacturer's instructions.

### Immunoblotting and immunohistochemical assay

Immunoblotting (IB) and Immunohistochemical (IHC) assays were performed as previously described [29]; the indicated antibodies are described in Supplemental Table s2.

### Terminal deoxynucleotidyl transferase dUTP nick end labeling assay

A terminal deoxynucleotidyl transferase dUTP nick end labeling (TUNEL) apoptosis detection kit (Millipore) was used according to the manufacturer’s instructions.

### Colony formation assay

The radiation sensitivity of cells was assessed by the colony formation test. Approximately 500 GSC cells transduced with different constructs were seeded into 6-well plates with irradiation doses of 0, 2, 4, 6, and 8 Gy. Two weeks later, the cells were fixed with methanol and stained with 0.5% crystal violet. Clones (> 50 cells) were counted by using a microscope, and the surviving fraction was calculated as the mean colony number/(cells inoculated × plating efficiency).

### BrdU comet assay

The bromodeoxyuridine labelling assay was conducted as described. Briefly, cultured single GSCs transduced with different constructs were pulse-labelled with 10 μM BrdU for 35 min and, subsequently subjected to irradiation at an indicated dose (5 Gy). Beoxynucleoside triphosphate (dNTP) pools were used to chase BrdU-labeled DNA at different time points: 0, 0.5, 2, 4, 8, and 12 h. Treated GSC Cell lysates were prepared, and electrophoresis was carried out under alkaline conditions, followed by immunoblotting with a specific anti-BrdU antibody to evaluate DNA migration. The tail moment was measured by Tritek Comet Score freeware v1.5 Software (Tritek Corp. Sumerduck, VA, USA).

### Apoptosis and cell cycle detection by flow cytometric assay

GSC cells following treatment were seeded in 6-well plates, and irradiated (5 Gy). Flow cytometry analysis was conducted according to previously reported procedures [30]. Apoptosis rates were detected by Annexin V-PI/FITC staining. Cell cycle distribution was analyzed by propidium iodide staining.

### Coimmunoprecipitation

Interactions between specific molecules were conducted via coimmunoprecipitation (Co-IP) assays, as previously described [31].

### Protein half-life assay

The indicated groups of GSCs were treated with 100 μg/ml CHX for 0, 3, 6, 9 h, or/and 12 h, followed by protein collection and western blotting to assess protein expression decay.

### GST pull-down assays

Recombinant GST-POLD4, GST-UCHL3-WT, and GST-UCHL3-S75A were cloned and inserted into the pGEX-4 T-1 bacterial expression plasmid, amplified in *Escherichia coli* strain BL21 under 0.4 mM isopropyl β-d-1-thiogalactopyranoside at 16 °C and obtained with GST beads (Sigma) following the standard instructions of manufacturer. For GST pull-down, Glutathione Sepharose beads coated with the indicated GST- fusion proteins (GST-POLD4, GST-UCHL3-WT, and GST-UCHL3-S75A) were incubated with HEK293T extract prepared from cells expressing Flag-UCHL3 WT, Flag-UCHL3 S75A, or His-POLD4 for 2 h at 4℃. After four washes in the binding buffer, bound proteins were eluted in SDS-sample buffer, boiled, subjected to SDS-PAGE, and blotted with the indicated antibody.

### In vivo and deubiquitylation assays of POLD4

For in vivo deubiquitylation assays of POLD4, 30 h posttransfection with the indicated plasmids, cells were challenged with the proteasome inhibitor MG132 (20 μM) for 6 h before cell harvest. Primary antibody (anti-POLD4) conjugated with protein A/G beads was incubated with the cell lysates overnight at 4 °C. Precipitates were boiled in SDS buffer, separated by SDS-PAGE, and probed by anti-HA immunoblotting.

For in vitro deubiquitylation assays of POLD4, HEK293T cells overexpressing His-tagged POLD4 and HA-tagged Ub were treated with MG132 (20 μM) for 6 h. The lysates from HEK293T cells were loaded onto a His-affinity column (Thermo Fisher Scientific) to obtain pure POLD4 with sufficient ubiquitination. Ubiquitylated POLD4 was incubated with recombinant protein (GST-UCHL3-WT or GST-UCHL3-S75A) in deubiquitination buffer (50 mM Tris–HCl pH 8.0, 50 mM NaCl, 1 mM EDTA, 10 mM DTT, 5% glycerol) for 2 h at 37 °C, and the resulting reactions were subjected to immunoblotting analysis.

### DUB screening for POLD4

siRNAs targeting DUBs from the Human siGenome SMART pool library (Dharmacon) were used in siRNA-mediated POLD4 silencing.

In short, specified amounts of HEK293T cells, according to the protocol, were seeded into custom 96-well plates in the presence of siRNA. Cells were subsequently lysed and analyzed by IB using the primary anti-POLD4 antibody.

### HR- or NHEJ-mediated DSB repair GFP reporter systems.

PDRGFP and pCBASceI were the gifts from Maria Jasin [32, 33](Addgene plasmid # 26475, plasmid # 26477), and pimEJ5GFP was a gift from Jeremy Stark [34](Addgene plasmid # 44026). For stable construction of cell lines, pCBASceI-stable transfectants in GSCs and HEK 293 T cells were selected using neomycin (600 μg/ml) for 2 weeks, and neomycin-resistant cells were cloned. Subsequently, pCBASceI-stable cell lines were transfected with PDRGFP or pimEJ5GFP, and cellular clones were generated by selection with puromycin (2 μg/ml) or ampicillin (100 μg/mL). Certain DR-GFP or EJ5-GFP reporter cell lines were transfected with the indicated constructs following IR. Two days later, the GFP + cells were assayed by FACScan.

### Statistical analysis

All experiments were repeated three times. Student’s *t*-test was performed for pairwise comparisons, whereas all datasets of ≥ three comparisons were analyzed with one-way analysis of variance (ANOVA). The Kaplan‐Meier method was applied to generate survival curves, and the log‐rank (Mantel‐Cox) test was used to determine statistical significance. *P*‐values of < 0.05 were considered statistically significant, and P‐values of < 0.005 were considered highly statistically significant.

## Results

### Overexpression of POLD4 corresponds to acquisition of the mesenchymal GSC phenotype and a poor prognosis in GBM

**Fig. 1:**
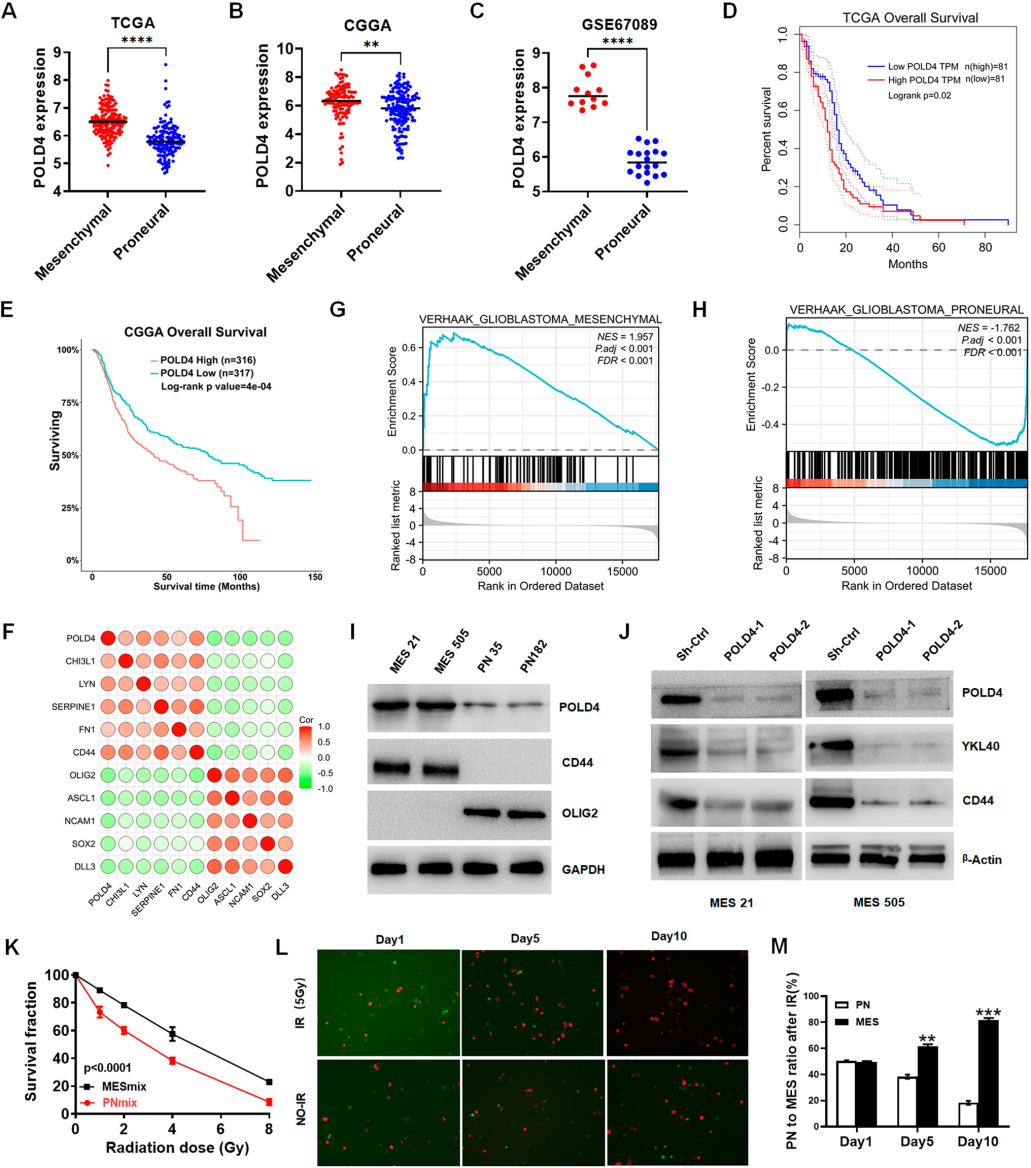
Overexpression of POLD4 corresponds to acquisition of the mesenchymal GSCs phenotype and a poor prognosis in GBM. **A**, **B**, **C** The expression of POLD4 mRNA mesenchymal (MES), and proneural (PN) phenotypes in TCGA GBM (**A**) and CGGA GBM (**B**) and GSE67089 (**C**). **D**, **E** Kaplan–Meier overall survival curves in TCGA GBM patients (**D**) and CGGA GBM patients (**E**) stratified based on POLD4 expression levels. **F** The association of POLD4 mRNA level with clusters identified markers: PN markers (e.g.DLL3, OLIG2) and MES markers (e.g.CD44, FN1). **G**, **H** POLD4 expression was analyzed between MES (**G**) and PN (**H**) signature and visualized with GSEA in TCGA. **I** IB analysis of POLD4, CD44, and OLIG2 in GCS cells (MES 21, 505; PN 35, 182). **J** IB analysis of POLD4, CD44, and YKL40 in MES GCSs with POLD4 knockdown. **K** Radiation survival curves of MES mix (MES 505 and MES 21) cells or PN mix (PN 35 and PN 182) cells after irradiation with 0-8 Gy. **L** MES and PN cells were labelled individually with CHERRY (red) and GFP (green) fluorescent dyes, mixed in equal ratios, exposed in IR and visualized by fluorescent microscopy at the particular time points. **M** Quantification of the percentage of MES and PN cells from (**L**), **P < 0.01, ***P < 0.001

To investigate the potential role of POLD4, we first surveyed the expression patterns of the POLD4 family in the publicly available GBM datasets CGGA and TCGA and performed survival analysis. The expression levels of POLD4 members (POLD1, POLD2, POLD3, POLD4) were significantly higher in GBM than in normal tissue in the TCGA dataset (Fig. S1A). In contrast, only POLD4 showed significant variation in expression across TCGA and CGGA datasets and mesenchymal subtype GBM with an elevated expression level (Fig. 1A, B and Fig. S1B). Similar results were obtained from the GSE67089 GSC dataset [4] analysis, where the expression level of POLD4 was substantially increased in the MES phenotype matched with the PN phenotype (Fig. 1C). In addition, We divided the patients into high and low groups according to the expression of POLD4 and evaluated the survival prognosis of GBM patients by Kaplan–Meier survival curve analysis, and the disease-free survival and overall survival of patients with high POLD4 expression was meaningly shorter both in both the CGGA and TCGA datasets than those with low expression. Interestingly, other POLD4 family members were unavailable to help guide the prognosis (Fig. 1D, E and Fig. S1C). Beyond this, we performed a gene-by-gene correlation analysis using the original sequence information extracted from the CGGA and TCGA databases of genotypes and observed that, in GBM cells, POLD4 expression correlated strongly with MES signature gene expression, but negatively correlated with PN gene marker expression (Fig. 1F and Fig. S1D). Furthermore, gene set enrichment analysis (GSEA) based on the TCGA dataset validated that enrichment of the MES subtype existed in the high POLD4 expression group, whereas enrichment of the PN subtype was present in the low POLD4 expression group (Fig. 1G, H). In addition to the transcriptional level, we detected POLD4 protein levels in patient-derived GSCs (MES21, 505 and PN35, 182) and found that POLD4 is overexpressed in MES GSCs but not in PN GSCs (Fig. 1I). We also tested the effect of altered POLD4 on GSC subtype markers and found that silencing POLD4 expression reduced MES marker protein levels and that POLD4 overexpression decreased PN marker intensity (Fig. 1J and Fig. S2A).

We have shown that PN and MES GSCs have different capacities for tumorigenicity and self-renewal [6]. To assess inherent differences in sensitivity to radiotherapy between PN and MES GSCs, we irradiated GSCs with varying doses of radiation and found that MES cells tended to be more resistant to radiation than PN GSCs (Fig. 1K and Fig. S2B). Concomitantly, induction of POLD4 protein accumulation by radiation was observed (Fig. S2C). Consistent with the observation of Fig. 1K, we noted a significant increase in the percentage of MES cells after treatment with IR (Fig. 1L, M). To determine whether the decreased cell numbers in GSCs were due to apoptosis, we analyzed GSCs for presence of activated caspase 3, a marker for cell death, and found that the preferential survival of MES cells after irradiation was due to lower rates of apoptosis, this was also confirmed by the lower expression of caspase-3 in irradiated MES GSCs than PN GSCs (Fig. S2D). In summary, these results suggest that POLD4 is involved in the regulation of MESGSCs, has stronger resistance to radiotherapy and predicts a poor prognosis.

### POLD4 enhanced the maintenance of GSC proliferation capacity and protection against radiation

**Fig. 2:**
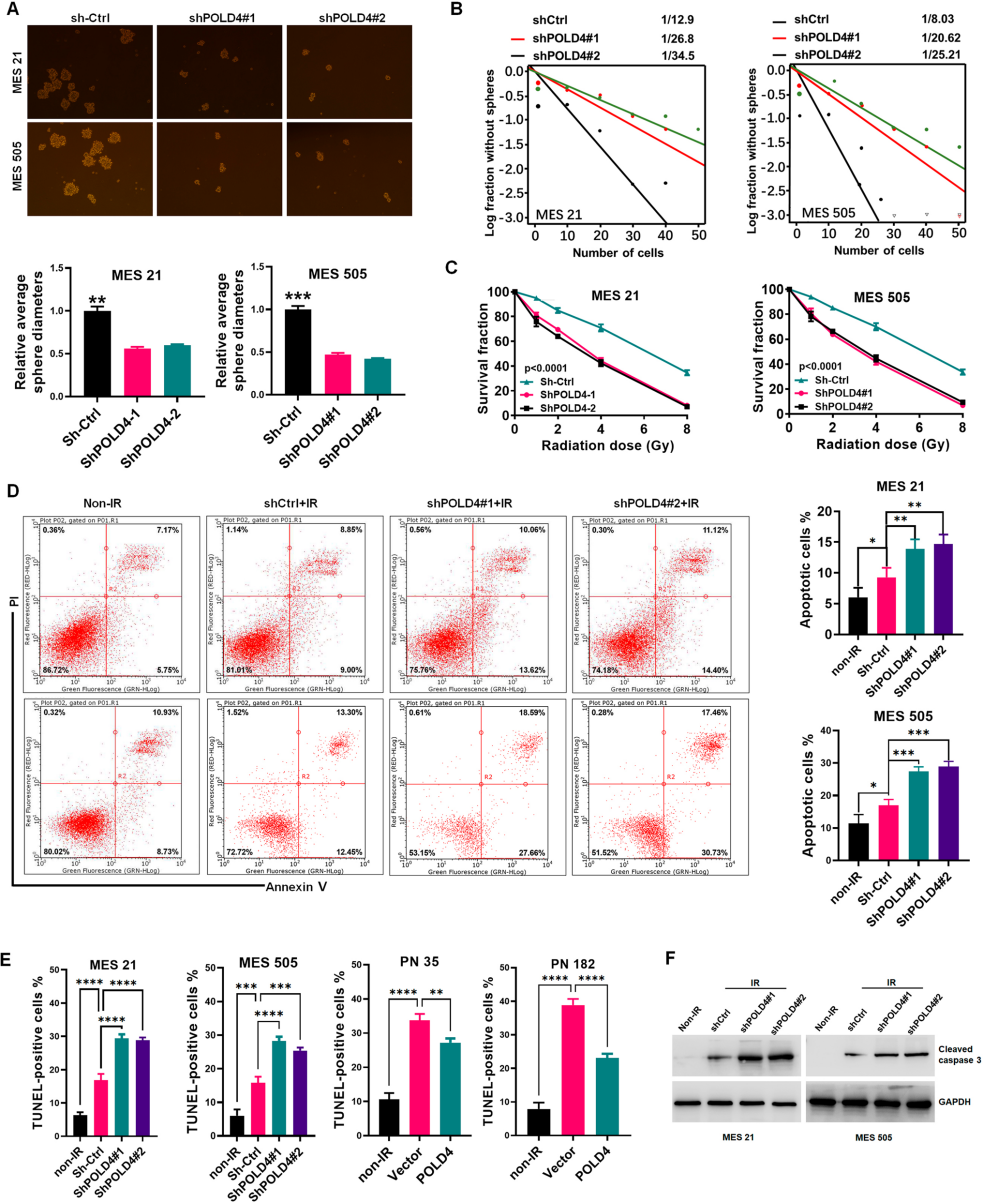
POLD4 enhanced maintenance of GSCs proliferation capacity and protection against radiation. **A** Representative neuro-sphere formation assay and quantitative analysis of MES GSCs transduced with non-silencing shRNA (shCtrl) or shPOLD4 (scale bar, 400 μm). **B** Limiting dilution assays of assay of MES GSCs transduced with shCtrl or shPOLD4. **C** Radiation survival curves of MES 505 and MES 21 transduced with shPOLD4 or shCtrl after irradiation with 0–8 Gy. **D** Apoptosis assay was analyzed and quantified by FACScan in MES 505 and MES 21 transduced with shPOLD4 or shCtrl, in response to IR (6 Gy). **E** TUNEL analysis of apoptosis in MES 505 and MES 21 (transduced with shPOLD4 or shCtrl) with or without irradiated (6 Gy). **F** IB analysis of cleaved caspase-3 in MES 505 and MES 21 (transduced with shPOLD4 or shCtrl) with or without irradiated (6 Gy). β-Actin served as control

**Fig. 3:**
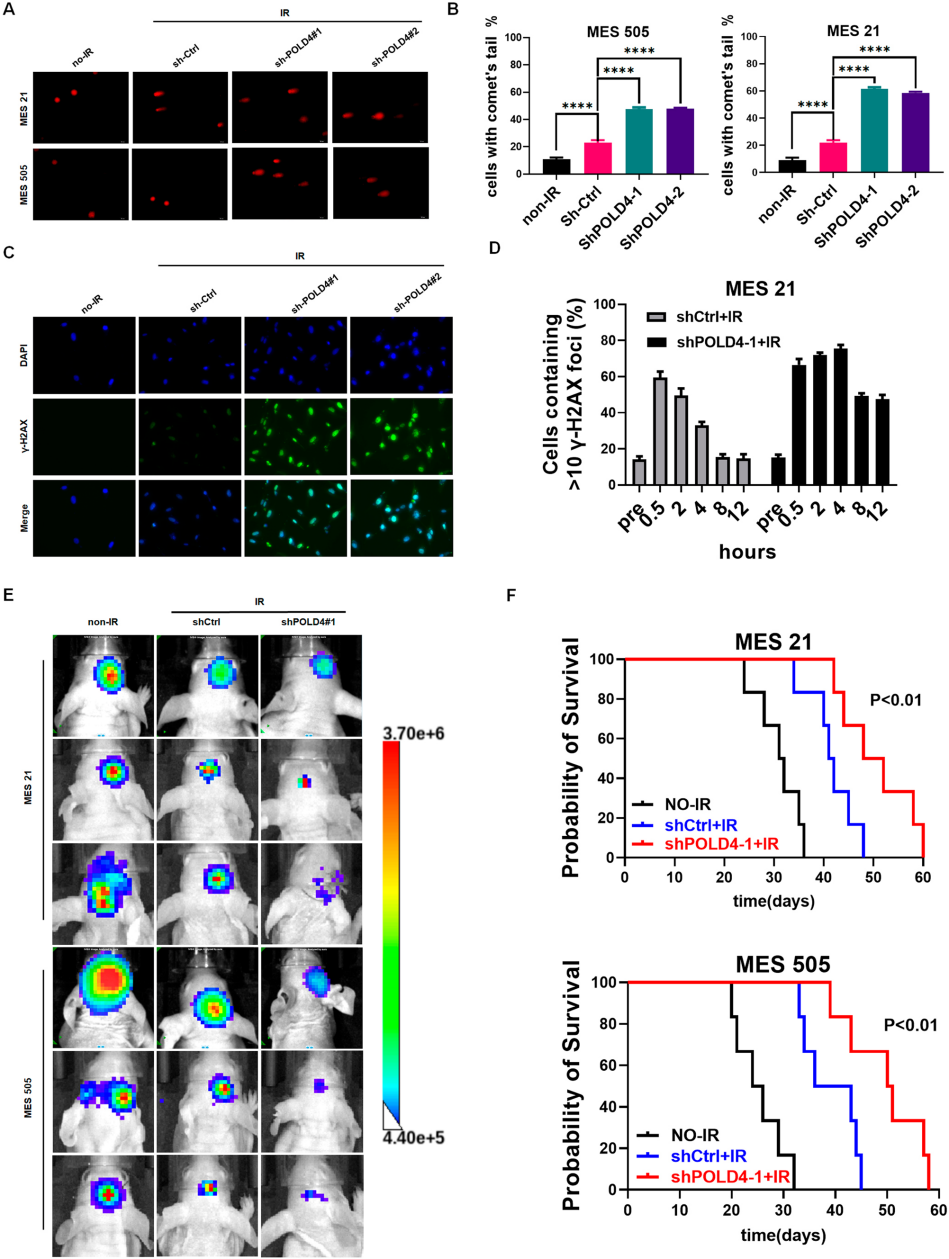
**A** Comet assays showed different levels of DNA damage in sh-POLD4 or shCtrl MES21 and MES505 cells with or without irradiated, Representative images are shown. **B** Quantification of Comet assays from (**A**). **C** Immunofluorescence analysis of γ-H2AX foci in sh-POLD4 or shCtrl MES21 with or without irradiated, Representative images are shown. **D** Quantification of γ-H2AX-foci-positive MES21 in different time points. **E** Representative bioluminescence images of xenograft tumors at day 28 after implantation of MES21or MES505 cells (transduced sh-POLD4 or shCtrl) with or without IR at 2.5 Gy daily for 4 days are shown. **F** Kaplan–Meier survival curve of mice intracranially implanted with MES 21 or MES 505 (expressed sh-Ctrl or sh-POLD4) with or without irradiated

Given the differential abundance of POLD4 in the GSC subtype and overexpression of POLD4 induced by IR in MES GSCs, to probe the effect of POLD4 on the biological functions of GSCs, we first performed a tumor sphere formation assay, limiting dilution assay. As expected, silencing of POLD4 considerably reduced MES cell sphere diameter, inhibited sphere formation, and also inhibited radiotherapy resistance in MES GSCs by colony formation assay (Fig. 2A, B, C), and overexpression of POLD4 exhibited the opposite effects in PN cells (Fig. S3A, B, C). Numerous studies have confirmed the possibility that IR induces PMT in GSCs [5, 35, 36]. IR has many effects on cells, including apoptosis, DNA breaks, and cell cycle arrest by checkpoints. Therefore, we designed a series of assays to assess the effect of POLD4 on GSCs in the context of radiation therapy. Apoptosis assays showed that following IR, MES cells indeed underwent apoptosis with accentuation due to silencing of POLD4, whereas apoptosis was curtailed by overexpression of POLD4 in PN cells (Fig. 2D and Fig. S3D). TUNEL assays yielded similar results (Fig. 2E). Meanwhile, knockdown of POLD4 meaningfully elevated caspase activation after irradiation (Fig. 2F). Furthermore, we sought to test the significance of POLD4 in the IR-induced DNA damage repair capacity of radioresistant GSCs. DNA damage was quantified using a comet assay and showed that depletion of POLD4 mitigated IR-induced DNA damage in MES GSCs. (Fig. 3A, B); however, PN GSCs with overexpressing POLD4 showed low sensitivity to IR (Fig. S4A). It is well-known that the rate of γ-H2AX decay after DNA injury is a sign of DNA double-stranded break (DSB) repair [37]. Based on this, we detected the immunofluorescence (IF) intensity of γ-H2AX to assess the effect of POLD4 on DNA damage-repair efficiency. The results showed that the knockdown of POLD4 affects the rate of γ-H2AX decay, resulting in DNA repair capacity with marked defects in MES GSCs (Fig. 3C, D, Fig. S4B, D). In contrast, PN GSCs overexpressing POLD4 overcame DNA damage induced by irradiation through efficient DNA damage repair (Fig. S4C, E). Moreover, IR generally caused an increased number of cells in G2/M, and we analyzed the effect of POLD4 on the G2/M checkpoint in GSCs after IR. MES GSCs indeed accumulated in the G2/M phase in response to IR as expected; surprisingly, pretreatment with shPOLD4 followed by IR resulted in a similar percentage of MES GSCs in the G2/M phase, suggesting that POLD4 may not play a decisive role in IR-induced G2/M arrest in GSCs (Fig. S4F, G). Finally, in addition to in vitro assays, we performed in vivo experiments in an orthotopic xenograft mouse model to assess the impact of POLD4 on IR resistance. Bioluminescence imaging test results revealed that mice bearing xenografts derived from MES GSCs followed by IR displayed a lower tumor formation rate and prolonged survival than those following no-IR treatment (Fig. 3E, F). If we implanted MES GSCs transduced with shPOLD4, the biological enhancement effects above were observed (Fig. 3E, F). Collectively, these results confirmed that POLD4 promotes GSC progression and radiotherapy resistance in vitro and in vivo.

### POLD4 protects MES GSCs against IR via the HR and NHEJ pathways

**Fig. 4:**
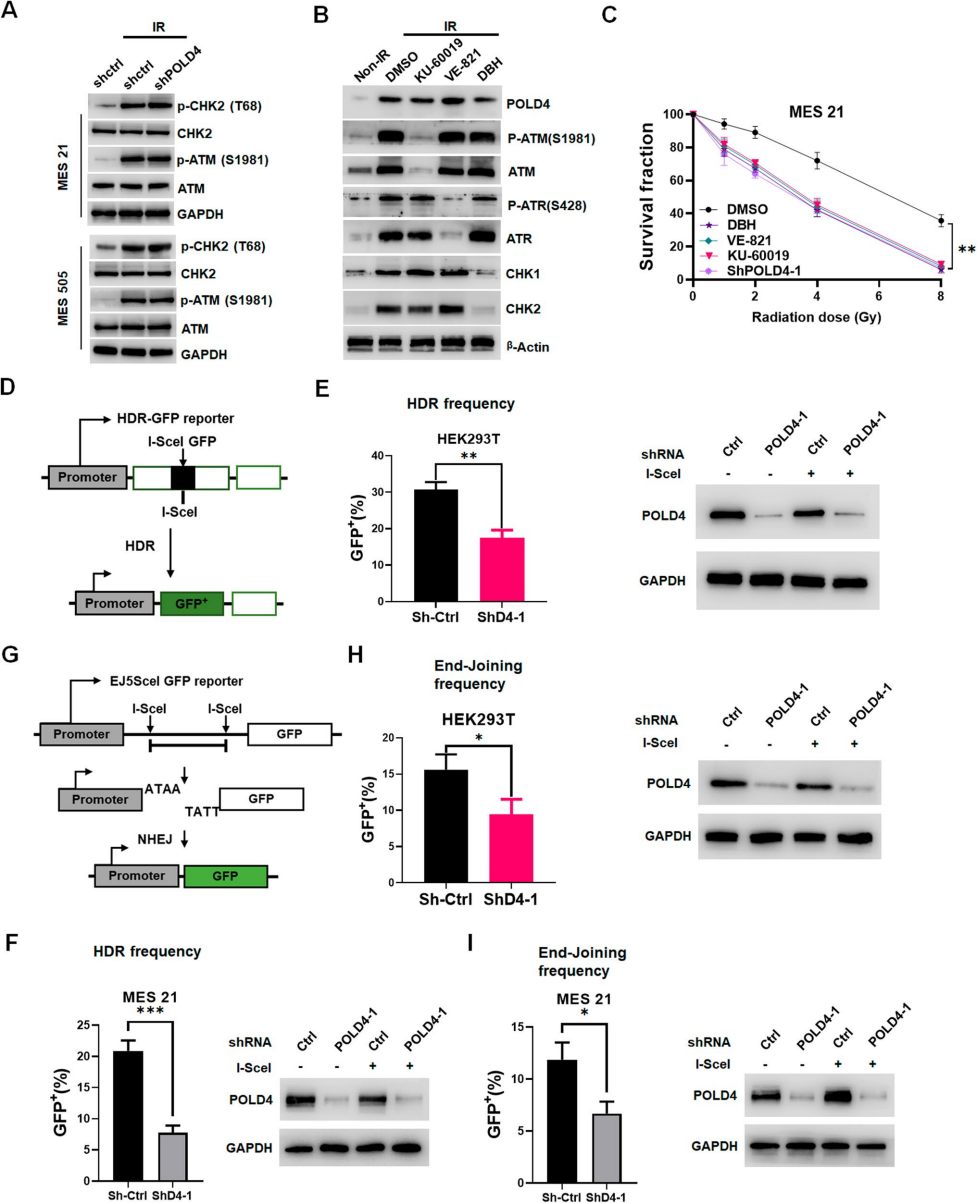
**A** IB analysis of p-ATM(S1981), p-Chk2 (T68), Chk2, ATM in MES 21 (top) and MES 505 (bottom) (transduced with shPOLD4 or shCtrl) with or without irradiated. **B** IB analysis of POLD4, P-ATM(S1981), P-ATR(S428), ATM, ATR, Chk1, Chk2 in MES 21 treated with DMSO, KU-60019 (selective ATM inhibitor), VE-821 (selective ATR inhibitor), DBH (CHK1/CHK2 inhibitor), in response to IR. **C** Radiation survival curves of MES 21 treated with DMSO, KU-60019, VE-821, DBH, shPOLD4 after irradiation with 0-8 Gy. **D** Schematic of the PDRGFP reporter assay. HDR of the I-SceI-induced DSB in SceGFP using eGFP as template leads to GFP expression. **E** PDRGFP stable HEK293T Cells were transfected with shPOLD4 or shCtrl. Cells were collected for flow cytometry and IB. **F** PDRGFP stable MES 21 were transfected with shPOLD4 or shCtrl. Cells were collected for flow cytometry and IB. **G** Schematic of the pimEJ5GFP reporter assay. The GFP cassette is split apart its promoter by two tandem I-SceI sites, such that EJ repair that uses distal ends of the two I-SceI-induced DSBs restores GFP expression. **H** pimEJ5GFP stable HEK293T Cells were transfected with shPOLD4 or shCtrl. Cells were collected for flow cytometry and IB. **I** pimEJ5GFP stable MES 21 were transfected with shPOLD4 or shCtrl. Cells were collected for flow cytometry and IB

DNA lesions mainly activate ATM/CHK2 and ATR/CHK1 signaling cascades, namely the DNA damage response (DDR), to stabilize the genome [38–40]. We wondered whether there was a preferential relationship between POLD4 and the signaling cascades above in GSCs. We found that IR did activate the pathways ATM/CHK2 and ATR/CHK1; however, depletion of POLD4 in MES GSCs or overexpression of POLD4 in PN GSCs had no effect on the expression of ATM/CHK2 and ATR/CHK1 (Fig. 4A and Fig. S5A, B). In turn, treatment with KU-60019 (selective ATM inhibitor), VE-821 (selective ATR inhibitor) or DBH (CHK1/CHK2 inhibitor) cannot change the expression of POLD4 in MES GSCs following IR (Fig. 4B and Fig. S5C). To demonstrate the biological effectiveness of these inhibitors, we analyzed the proliferation of MES GSCs treated with these inhibitors and shPOLD4. The data showed that KU-60019, WE-821, DBH and silencing POLD4 suppressed MES GSC growth in combination with radiotherapy (Fig. 4C and Fig. S5D). In addition to the “conventional” DNA repair pathway, POLD4 might be implicated in the acquisition of salvage pathways. There have been reports that for DSBs, the two major conservative DSB repair pathways, NHEJ and HR, are dominant [41]. The replication stress response defect is induced by overexpression of oncogenes in common cell lines, converting to stem-like cells [42]. Together, these results highlight the complexity of the mechanisms that coordinate DNA damage repair in GSCs. This inspired us to explore the effect of POLD4 on HR or NHEJ repair efficiency with well-established HR and NHEJ assays in MES GSCs. For the HDR-GFP reporter assay, the use of the downstream internal GFP sequence (iGFP) as a template can repair a DSB resulting from the induction of I-SceI cleavage and produce GFP-positive cells (Fig. 4D). In HEK293T cells, POLD4 knockdown significantly decreased HDR efficiency (Fig. 4E). Because DNA-PK and RAD51 are important participants in NHEJ, the expression levels of both were detected after POLD4 knockdown, and the expression levels of DNA-PK and RAD51 were decreased (Fig. S5E). Furthermore, depletion of POLD4 reduced HDR efficiency in MES GSCs (Fig. 4F), and overexpression of POLD4 increased HDR efficiency in PN GSCs (Fig. S5F). For the NHEJ reporter assay, a GFP cassette is separated from its promoter by a marker gene that is flanked by two tandem I-SceI sites, such that EJ repair that uses distal ends of the two I-SceI-induced DSBs restores GFP expression (Fig. 4G). In HEK293T, POLD4 knockdown significantly decreased NHEJ repair efficiency (Fig. 4H). Further, depletion of POLD4 reduced NHEJ repair in MES GSCs (Fig. 4I), and overexpression of POLD4 increased NHEJ repair efficiency in PN GSCs (Fig. S5G).

### UCHL3 interacted with, stabilized and depolyubiquitylated POLD4

**Fig. 5:**
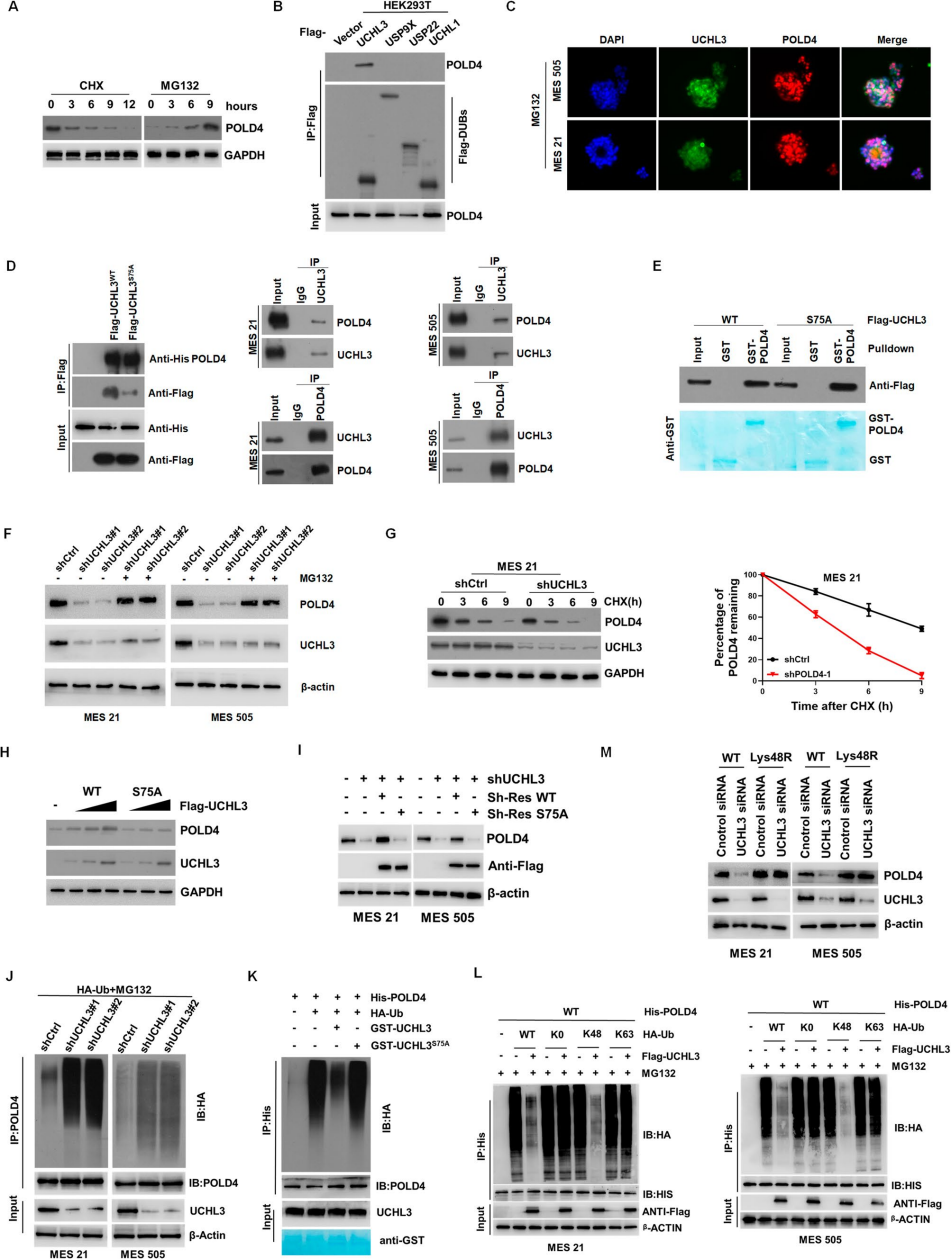
**A** The protein levels of POLD4 in HEK293T treated with 100 μg/ml CHX or 20 μM MG132 for indicated time points by IB analysis. **B** Lysates from 293 T cells transfected with Flag-tagged DUBs (UCHL1, UCHL3, USP9X, USP22) were subjected to IP with Flag beads, followed by IB with the indicated antibodies (POLD4 and Flag). **C** Co-IF staining assay showing the subcellular distribution of POLD4 with UCHL3 in MES21 and MES 505. Scale bar, 5 μm. **D** For 293 T cells, lysates from cells transfected with His-POLD4 alone or along with Flag-tagged UCHL3 WT or S75A mutant were subjected to IP assay with Flag beads, followed by IB with the indicated antibodies (His and Flag); For MES21 and MES 505, lysates from cells were subjected to IP analysis using anti- POLD4 antibody or anti-UCHL3 antibody and IB analysis, IgG isotype served as the isotype control. **E** GST or GST fusion proteins (GST-POLD4) were incubated with purified Flag-proteins (UCHL3 or S75A mutant) and the bound proteins were analyzed by SDS-PAGE, followed by Coomassie blue staining for GST proteins (bottom panels) or IB analysis with anti-Flag antibody (top panels). **F** IB analysis of POLD4 and UCHL3 protein levels in MES 21 and 505 GSCs transfected with 2 independent shUCHL3 or shctrl in presence of MG132 or not. **G** MES 21 stably expressing shUCHL3 or shctrl were treated with CHX (0.1 mg/ml) and harvested at the indicated time points for IB analysis of POLD4 and UCHL3 protein levels. For POLD4 decay, quantification of POLD4 levels relative to β-actin is shown. **H** HEK293T cells were transfected with either an empty vector or increasing amount of FLAG-tagged UCHL3 (WT or S75A), lysates from cells were subjected to IB analysis using anti-POLD4 antibody or anti-UCHL3 antibody. **I** Endogenous USP9X expression was stably knocked down, then transfected with either shRNA-resistant Flag-tagged UCHL3 WT or S75A mutant; lysates from cells were subjected to IB analysis using anti-POLD4 antibody or anti-UCHL3 antibody. **J** Lysates from MES 21 and 505 GSCs cotransfected with shUCHL3 or shctrl and HA-Ub were subjected to IP using POLD4 antibody followed by IB analysis with anti-POLD4 or anti-HA antibodies. Proteasome inhibitor (MG132, 20 μM) was added 8 h before cell collection. **K** GST fusion proteins (GST-UCHL3 WT or GST-UCHL3 S75A) were incubated with unubiquitylated or ubiquitylated His-POLD4 and the bound proteins were analyzed by SDS-PAGE, followed by Coomassie blue staining for GST proteins or IB analysis with anti-HA or anti-His antibody. **L** Lysates from MES 21 and 505 GSCs cotransfected with His-POLD4, Flag-UCHL3 and HA-Ub (wt), HA-Ub (K48), or HA-Ub (K63) were subjected to IP using anti-His antibody followed by IB analysis with anti-His or anti-HA antibodies. Proteasome inhibitor (MG132, 20 μM) was added 8 h before cell collection. **M** IB analysis of POLD4 and UCHL3 protein levels in MES 21 and 505 GSCs transfected with HA-Ub (wt) or HA-Ub (K48) in the presence of shctrl or shUCHL3

POLD4 is a short-lived protein, whose abundance is frequently determined by posttranslational modifications (PTMs) including ubiquitin-mediated proteolysis [43, 44]. To identify whether the protein expression of POLD4 is posttranslationally regulated by the ubiquitin–proteasome system (UPS) in GSCs and HEK293T cells, we performed a quantitative cycloheximide (CHX) chase assay to specifically monitor the rate of POLD4 degradation and found that the POLD4 protein levels gradually decreased after CHX treatment and were essentially undetectable by 12 h (Fig. 5A, left). Subsequently, the rapid degradation of POLD4 was prevented by MG132 treatment (Fig. 5A, right). These results verified that POLD4 stability is controlled by proteasomal degradation. We carried out a systematic DUB RNAi screen using a commercially available RNAi library and identified 4 candidate DUBs (UCHL1, UCHL3, USP9X, USP22) for the regulation of POLD4 (Fig. S6A, B). Furthermore, to determine which DUB interacts directly with POLD4, we performed coimmunoprecipitation experiments by overexpressing tagged DUBs in HEK293T cells, and showed that the candidate DUBs (except UCHL3) were not capable of directly interacting with the endogenous POLD4 (Fig. 5B). Additionally, fluorescence staining showed that UCHL3 and POLD4 partially colocalized to the nucleus (Fig. 5C). Then, we confirmed these interactions by co-IP assay and GST pull-down assay and found that inactive UCHL3 (Flag-UCHL3 S75A) can bind POLD4 directly, indicating that the enzyme activity of UCHL3 was not crucial for their interaction (Fig. 5D, E).

A reciprocal relationship between UCHL3 and POLD4 was confirmed, and we determined the clinical correlations and regulatory mechanisms of UCHL3 and POLD4. We first surveyed the expression patterns of UCHL3 in the TCGA and GSE67089 GSC datasets, where the expression level of UCHL3 was substantially increased in MES subtype GSCs as opposed to PN subtype GSCs (Fig. S6C, D). In addition, we performed Kaplan–Meier survival curve analysis to evaluate the prognosis of UCHL3 expression in TCGA, and the disease-free survival and overall survival of patients with high UCHL3 expression were shorter than those of patients with low UCHL3 expression (Fig. S6E). Using immunohistochemical staining, we expanded the analysis of UCHL3 and POLD4 protein expression in GBM clinical tumor tissues, and a substantially positive correlation was presented (Fig. S6F, G). Clinically, the high expression of UCHL3 and POLD4 protein in GBM is related to a worse patient prognosis (Fig. S6H, I). In addition to GBM, we detected the protein levels of patient-derived GSCs (MES21, 505 and PN35, 182) and found that UCHL3 was overexpressed in MES GSCs but not in PN GSCs (Fig. S6J).

Given the well-known function of UCHL3 as a deubiquitinating enzyme catalyzing the reversal of ubiquitination of target proteins, we proposed that UCHL3 could regulate POLD4 degradation in a deubiquitination-dependent manner. Indeed, we found that silencing UCHL3 reduced POLD4 protein expression (not mRNA), which was reversed by the UPS inhibitor MG132 (Fig. 5F and Fig. S6K). The CHX chase assay showed that depletion of UCHL3 decreased the rate of POLD4 degradation in MES GSCs (Fig. 5G and Fig. S6L). Overexpression of wild-type UCHL3 (WT-UCHL3), but not the catalytically dead mutant S75A, induced elevated POLD4 levels in a dose-dependent manner in HEK293T cells (Fig. 5H), implying that the enzyme activity of UCHL3 was crucial for stable POLD4. Only sh-Res UCHL3-WT, but not sh-Res UCHL3-S75A, restored POLD4 expression downregulated by shUCHL3, highlighting the importance of the enzyme activity of UCHL3 (Fig. 5I).

In view of the essential role of the enzyme activity of UCHL3 in driving POLD4 stabilization, we proposed that UCHL3 may deubiquitylate POLD4. In MES GSCs, knockdown of UCHL3 increased the levels of POLD4 ubiquitination (Fig. 5J). To directly detect the deubiquitination ability of UCHL3 toward POLD4, we carried out an in vitro deubiquitination assay and found that the UCHL3 wild-type (WT), but not the S75A mutant, deubiquitinated polyubiquitinated POLD4 (Fig. 5K). Different types of ubiquitin linkages endow target proteins with functional versatility. We tried to determine which ubiquitin linkage type of POLD4 participates in the regulation of UCHL3. Co-IP results showed that in wild-type (WT) and a range of ubiquitin lysine (K) mutants, K48, K63, and K0 (a ubiquitin mutant with all K residues replaced by R), UCHL3 removed the K48-type linkage of the ubiquitin chain of POLD4, but had no effect on other mutant-linked ubiquitylation of POLD4 in MES GSCs (Fig. 5L). Stabilization of POLD4 assays revealed that ectopic expression of K48R (not WT) ubiquitin restored the POLD4 expression inhibited by UCHL3 depletion in MES GSCs (Fig. 5M).

### UCHL3 promotes GSC progression and IR resistance via POLD4

**Fig. 6:**
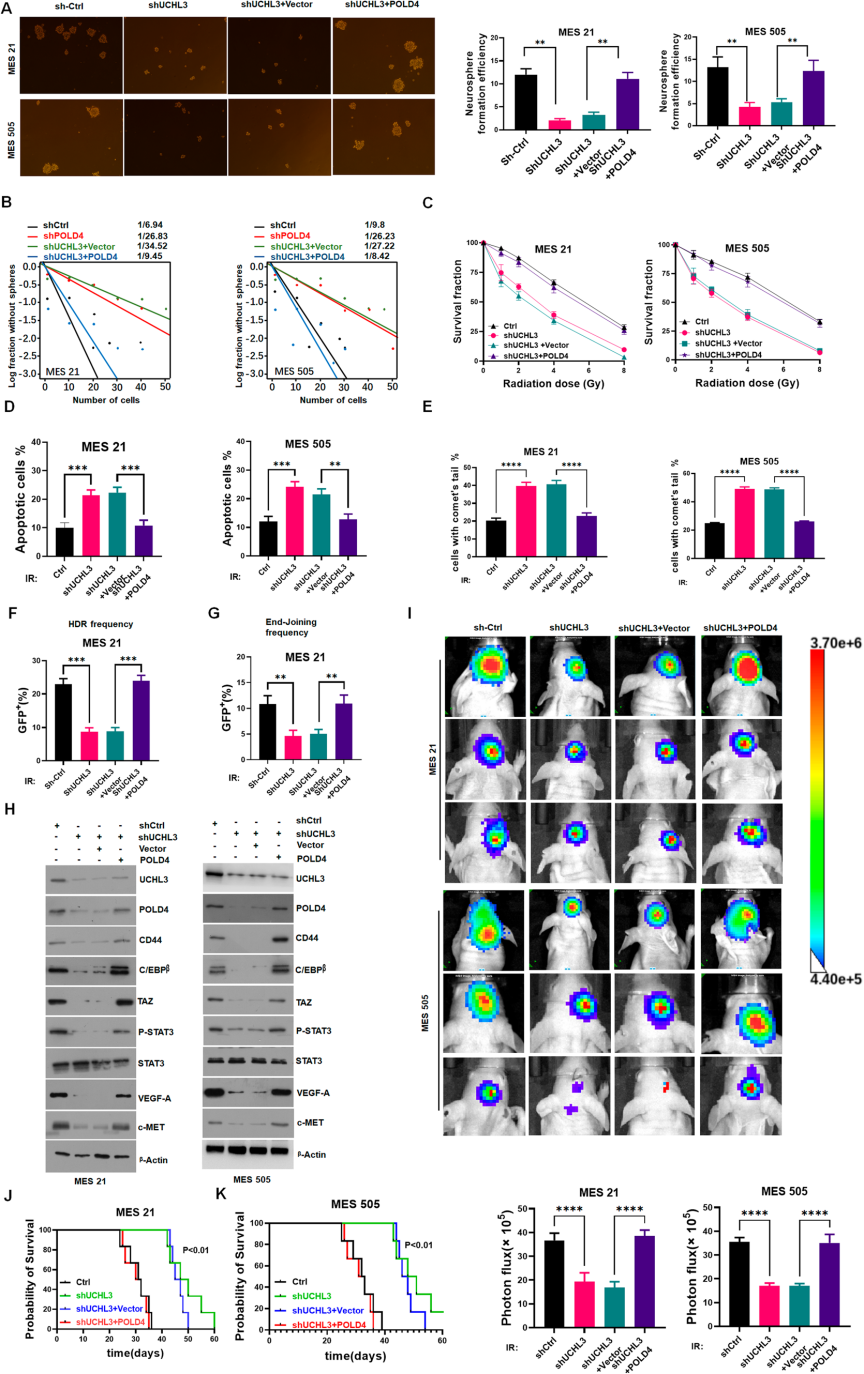
**A** Representative images and the quantification of neuro-sphere formation assay in MES 21 and MES 505 transduced with control non-silencing shRNA (shCtrl) or shUCHL3, along with Vector or POLD4. **B** Limiting dilution assays of assay in MES 21 and MES 505 transduced with control non-silencing shRNA (shCtrl) or shUCHL3, along with Vector or POLD4. **C** Radiation survival curves of MES 505 and MES 21 transduced with shCtrl or shUCHL3, along with Vector or POLD4 after irradiation with 0–8 Gy. **D** Apoptosis assay was analyzed and quantified by FACScan in MES 505 and MES 21 transduced with shCtrl or shUCHL3, along with Vector or POLD4, in response to IR (6 Gy). **E** Comet assays showed different levels of DNA damage in MES 505 and MES 21 transduced with shCtrl or shUCHL3, along with Vector or POLD4 in response to IR (6 Gy). **F** PDRGFP stable MES 21 were transfected with shCtrl or shUCHL3, along with Vector or POLD4, and then exposed to IR(6 Gy). Cells were collected for flow cytometry. **G** pimEJ5GFP stable MES 21 were transfected with shCtrl or shUCHL3, along with Vector or POLD4, and then exposed to IR(6 Gy). Cells were collected for flow cytometry. (H) IB analysis of UCHL3, POLD4, CD44, C/EBPβ, TAZ, p-STAT3, STAT3, VEGF-A, and c-MET levels in MES 21 and MES 505 transfected with shCtrl or shUCHL3, along with Vector or POLD4. **I** Representative bioluminescence images and the quantification of tumor photon counts of xenograft tumors at day 28 after implantation of MES21 or MES505 GSC cells (transduced with shCtrl or shUCHL3, along with Vector or POLD4) after IR at 2.5 Gy daily for 4 days are shown. **J**, **K** Kaplan–Meier survival curve of mice intracranially implanted with MES 21 (**J**) or MES 505 (**K**) expressing shCtrl or shUCHL3, along with Vector or POLD4 after IR

UCHL3, as a bona fide deubiquitinase of POLD4, was identified in MES GSCs. Next, we propose that UCHL3 regulates the biological function of POLD4 in MES GSCs. Tumor sphere formation assays and limiting dilution assays showed that POLD4 overexpression reversed the effects of UCHL3 on the sphere-forming ability and self-renewal capacity of MES GSCs (Fig. 6A, B). Colony formation assays indicated that POLD4 overexpression restored the proliferation ability reduced by UCHL3 depletion in the case of IR (Fig. 6C). Furthermore, under conditions of IR, the level of apoptosis increased by silencing UCHL3 was overcome by POLD4 overexpression in MES GSCs (Fig. 6D and Fig. S7A). A comet assay showed that depletion of UCHL3 increased IR-induced DNA damage in MES GSCs, but POLD4 overexpression counteracted the effect of UCHL3 knockdown (Fig. 6E). For the HDR and NHEJ reporter assay, silencing UCHL3 reduced HDR and NHEJ efficiency in MES GSCs followed by IR, and ectopic expression of POLD4 overcame the inhibitory effects of UCHL3 depletion on MES GSCs (Fig. 6F, G and Fig. S7B, C). At the molecular level, under IR exposure UCHL3 knockdown reduced the protein expression of well-known MES GSC markers (CD44, TAZ, etc.), and POLD4 overexpression restored the expression of these markers (Fig. 6H). Finally, mice bearing xenografts derived from MES GSCs treated with joint UCHL3 depletion and IR displayed a lower rate of tumor formation and prolonged survival compared to those following IR treatment, and POLD4 overexpression inhibited this synergism (Fig. 6I, J, K). Collectively, UCHL3 enhances the self-renewal, proliferation, tumorigenesis and IR resistance of GSCs by regulating POLD4.

### UCHL3 inhibitor TCID increases MES GSC radiosensitivity

**Fig. 7:**
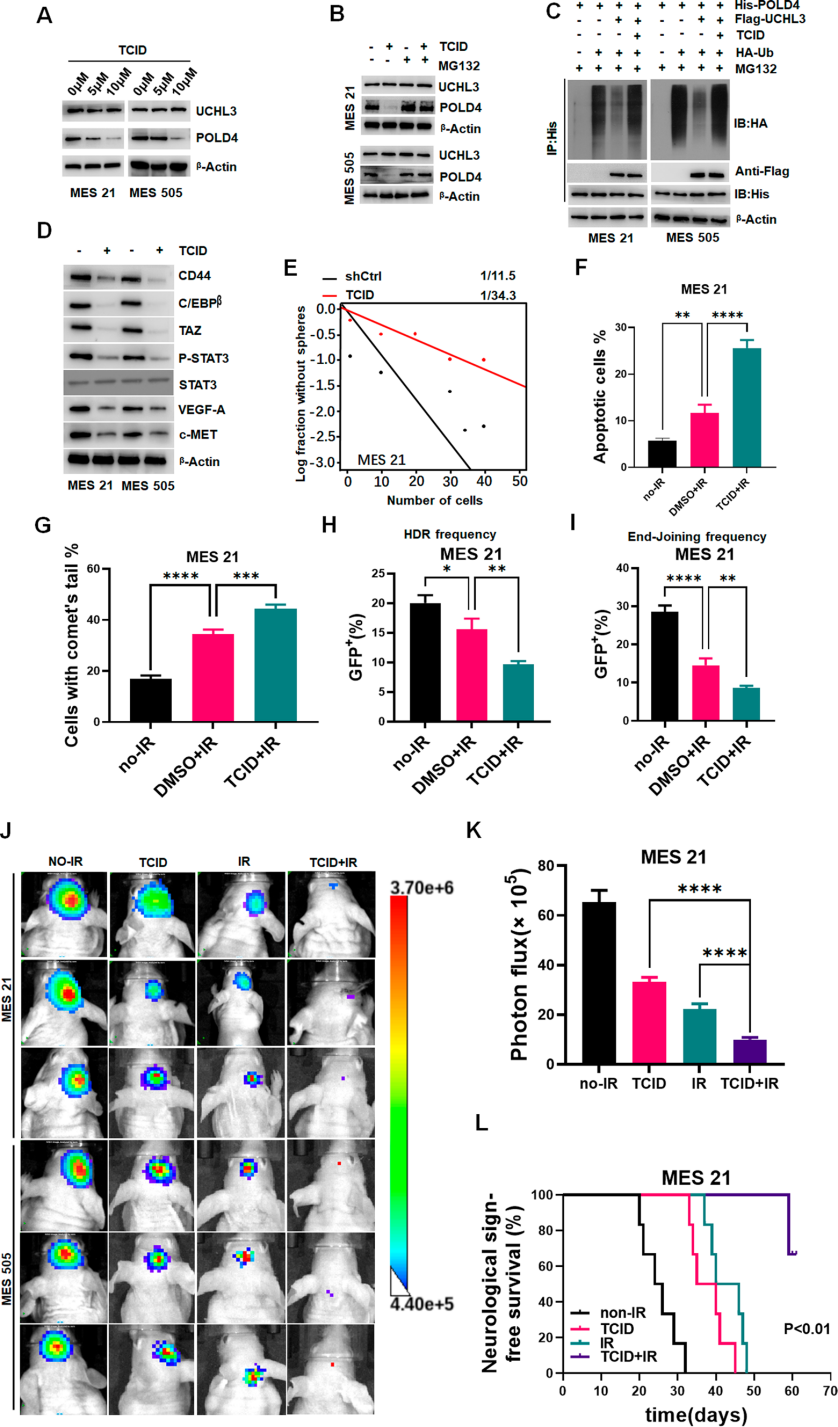
**A** IB analysis of POLD4 protein expression in MES 21 and MES 505 treated with TCID in indicated concentrations. **B** IB analysis of POLD4 in MES 21 and 505 GSCs treated with 10 μM TCID or vehicle in present of MG132 or not. **C** Lysates from MES 21 and 505 GSCs cotransfected with His-POLD4, Flag-UCHL3 and HA-Ub in the absence or presence of 10 μM TCID were subjected to IP using anti-His antibody followed by IB analysis with anti-His or anti-HA antibodies. Proteasome inhibitor (MG132, 20 μM) was added 8 h before cell collection. **D** IB analysis of CD44, C/EBPβ, TAZ, p-STAT3, STAT3, VEGF-A, and c-MET levels in MES 21 and 505 GSCs treated with 10 μM TCID or vehicle. **E** Limiting dilution assays of assay in MES 21 treated with 10 μM TCID or vehicle. **F** Apoptosis assay was analyzed and quantified by FACScan in MES 21 treated with 10 μM TCID or vehicle after IR (6 Gy). **G** Comet assays showed different levels of DNA damage in MES 21 treated with 10 μM TCID or vehicle after IR (6 Gy). **H** PDRGFP stable MES 21 treated with 10 μM TCID or vehicle after IR (6 Gy). Cells were collected for flow cytometry. **I** pimEJ5GFP stable MES 21 treated with 10 μM TCID or vehicle after IR (6 Gy). Cells were collected for flow cytometry. **J** Representative bioluminescence images of xenograft tumors at day 28 after implantation of MES21 or MES505 after indicated treatment are shown (IR dose of 2.5 Gy per day for four consecutive days). **K** The quantification of tumor photon counts on day 28 after MES 21 intracranial transplantation for mice with indicated treatment. **l** Kaplan–Meier survival curves of mice bearing MES 21-derived xenografts treated with the indicated interventions

Sundry DUBs are charming pharmaceutical targets, with some DUB inhibitors recently in preclinical development [45]. To exploit the clinical implications of the data presented above, we introduced TCID, a well-established and selective UCHL3 inhibitor [28, 46], to our study. MES GSCs were treated with TCID at serial concentrations, and the appropriate final concentration was 10 μmol as the protein expression of POLD4 was almost depleted without affecting UCHL3 protein level (Fig. 7A). MG132 restored POLD4 expression reduced by TCID, implying that TCID suppressed POLD4 through ubiquitination in MES GSCs (Fig. 7B). Subsequently, the results confirmed that TCID blocked the capacity of MES GSCs to hydrolyze ubiquitin from POLD4 (Fig. 7C). TCID also suppressed the protein expression of MES GSC markers (Fig. 7D). In MES GSCs, TCID considerably reduced MES cell sphere formation and growth (Fig. 7E and Fig. S8A, B). TCID synergized with IR to induce the MES cell apoptosis rate (Fig. 7F and Fig. S8C), and aggravate DNA damage in MES GSCs (Fig. 7G and Fig. S8D). TCID inhibited the HDR and NHEJ frequency followed by IR (Fig. 7H, I). Tumours in mice treated with TCID and IR grew more slowly and mice survived longer than those treated with control mice, and we found that the combination of the two was more effective than either TCID or IR alone. (Fig. 7J, K, L, and Fig. S8E, F). Collectively, our results may play an important role in the design of potential drugs in clinical trials.

## Discussion

Radiotherapy is a critical component of GBM treatment, but radioresistance due to ill-defined mechanisms remains a major limitation resulting in GBM relapse and lethality [47]. Modalities that GBM uses to develop radioresistance include heterogeneity of GBM cells, PMT modulation in GSCs, acquisition of salvage pathways, and enhanced DNA repair [48]. Focusing on these modalities is promising for optimizing GBM therapy. In the present study, we determined that POLD4 confers a GSCs MES phenotype and was associated with dismal clinical outcomes in GBM patients. Based on a DUB-siRNA profiling screen, UCHL3, as a unique DUB of POLD4, was confirmed to bind directly to, stabilizes, and deubiquitinates POLD4 in GSCs. Meanwhile, the data from tissue specimens verified the positive correlation between UCHL3 and POLD4. Regulating biological functions, UCHL3 promoted GSC proliferation, tumorigenesis, self-renewal, and radioresistance in a POLD4-dependent manner.

The degradation of POLD4 by ubiquitin ligases in response to DNA damage and during cell cycle progression plays a less prominent role in normal cells [43], while with increasing insights into the complex mechanisms of DNA damage repair, POLD4 is projected to play an increasingly important role. For example, Huang QM et al. reported that a defect in p12 (namely, POLD4) was also detected in a subgroup of non-small cell lung cancer patients and was correlated with poorer prognosis. Zhang et al. demonstrated that p12 KO cells exhibit increased sensitivity to chemotherapeutic agents and synthetic lethal sensitivity to PARP inhibitors [14]. Our findings showed that POLD4 directly enhances the radiation resistance of GSCs through heightened DNA damage repair by HR and NHEJ. Routinely, DNA damage leads to the activation of stress responses in coordination with cell cycle arrest, and the integrity of genomes is maintained. Meanwhile, DNA damage contributes to the construction of abnormal DNA structures that activate the DDR signaling pathway including ATM/CHK2 and ATR/CHK1. We proved that POLD4 was not correlated with ATM/CHK2 and ATR/CHK1, and loss-of or gain-of function of POLD4 did not affect the GSC cell cycle (Fig. S4F, G), suggesting that HR and NHEJ are additional mechanisms for radiosensitization mediated by POLD4 in GSCs (Fig. 4F, I and Fig. S5E, F). McGrail et al. demonstrated that a defective replication stress response is inherently linked to the cancer stem cell phenotype [42].

Several DUBs have been directly implicated in GSC PMT marker deubiquitination. USP21 promotes self-renewal and tumorigenicity of mesenchymal glioblastoma stem cells by deubiquitinating and stabilizing FOXD1 [49]. We identified USP9X as a bona fide deubiquitinase of ALDH1A3 in MES GSCs [6]. UCHL3, which is a deubiquitinating enzyme of the UCH family, plays a role in a variety of biological processes, including differentiation, proliferation, apoptosis, and progression of tumors. Pu et al. reported that UCHL3 promotes bone marrow mesenchymal stem cell osteogenesis by targeting SMAD1 [50]. Song et al. revealed that UCHL3 regulates the ubiquitination and stabilization of FOXM1, thereby potentiating pancreatic cancer progression and chemoresistance [46]. Zhang et al. found that UCHL3 promotes ovarian cancer progression by stabilizing TRAF2 to activate the NF-κB pathway [51]. Li et al. showed that UCHL3 regulates SOX12 via the AKT/mTOR signaling pathway and facilitates tumor progression [24]. Nishi showed that depleting UCHL3 resulted in reduced Ku80 foci formation, Ku80 binding to chromatin after DSB induction, moderately sensitized cells to ionizing radiation and decreased NHEJ efficiencies[52]. Luo et al. identified that UCHL3 is vital for HR and radioresistance in breast cancer [53]. These results prompt us that UCHL3 is a potential link between radioresistance and PMT in GSCs. Not coincidentally, in this study, we used a DUB-siRNA profiling screen deliberately identify UCHL3, which interacted with, deubiquitylated, and stabilized POLD4. UCHL3 promotes PMT in GSCs and IR resistance via POLD4.

Currently, small-molecule drugs targeted specifically against POLD4 have not been developed clinically. Promisingly, DUBs have become prominent targets for agent development to confront tumors, with DUB antagonists now progressing into preclinical tests or clinical trials [54, 55]. TCID attenuated UCHL3 activity without affecting its protein level and diminished tumor stem-like properties [28]. Focusing on mesenchymal targeting through UCHL3 inhibition (TCID), we found that triggering PMT by TCID in GSCs elevates their sensitivity to IR.

Temozolomide combined with radiotherapy is the main treatment for glioma after surgery, in this study, we experimentally demonstrated that TCID, an inhibitor of UCHL3, has a specific synergistic effect on treatment, and the combination of the two can inhibit the growth of tumour to a greater extent. However, we didn't design an experiment to explore the relationship between TCID and temozolomide in this study, and in the subsequent research plan, we will have an in-depth study on the relationship between the two. On the other hand, in this study, no signs of toxicity of TCID were detected after monitoring the vital signs and daily activities of mice treated with TCID. Considering the limited number of samples, the specific toxicological properties of TCID need to be further verified.

In summary, this study identifies UCHL3 as an indispensable deubiquitinase for stabilizing POLD4, which results in the acquisition of MES characteristics, IR resistance and tumorigenic ability in GSCs. Administering the expression of POLD4 via pharmacological suppression of UCHL3 may thus open up a novel route to therapeutic strategies in GBMs.

### Supplementary Information

Below is the link to the electronic supplementary material.

Supplementary file1 (DOCX 3625 KB)

## Data Availability

The datasets used and/or analyzed in the current study are available from the corresponding author on reasonable request.
