# Supplementary material for: UCHL3 induces radiation resistance and acquisition of mesenchymal phenotypes by deubiquitinating POLD4 in glioma stem cells
Source: Cell Mol Life Sci. 2024 Jun 3;81(1):247. doi: 10.1007/s00018-024-05265-5 (PMC11149539; doi:10.1007/s00018-024-05265-5)
Supplement: Supplementary file 1 — Supplementary file1 (DOCX 3625 KB) [file 18_2024_5265_MOESM1_ESM.docx]

**Figure S1**

**
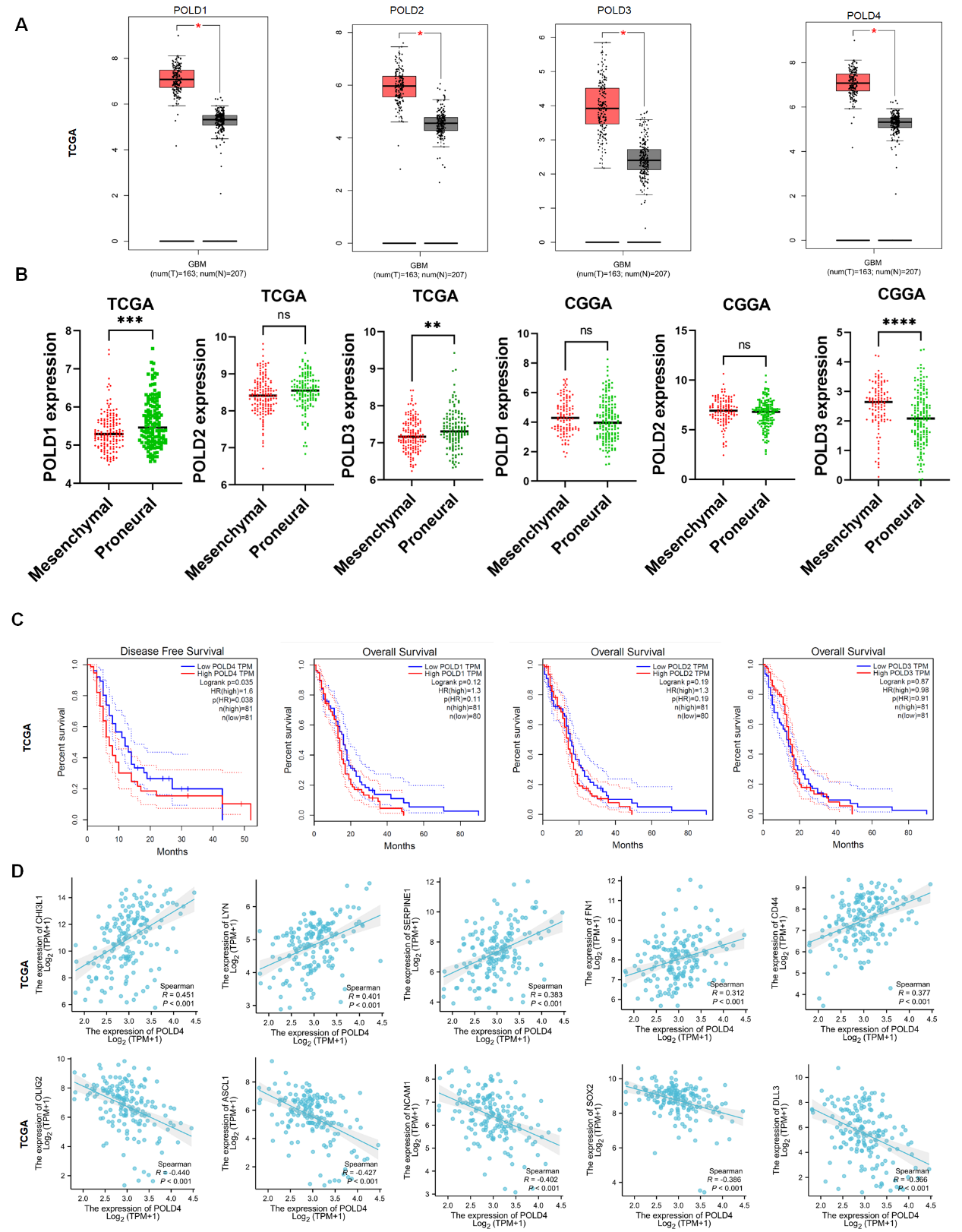
**

Figure S1 (A) The expression of POLD (1-4) mRNA in TCGA GBM. (B) The expression of POLD (1-3) mRNA mesenchymal (MES), and proneural (PN) phenotypes in TCGA GBM and CGGA GBM. (C) Kaplan-Meier overall or disease-free survival curves in TCGA GBM patients based on POLD (1-4) expression levels. (D) Correlation analysis of POLD4 with MES markers and PN markers in TCGA-GBM.

**Figure S2**

**
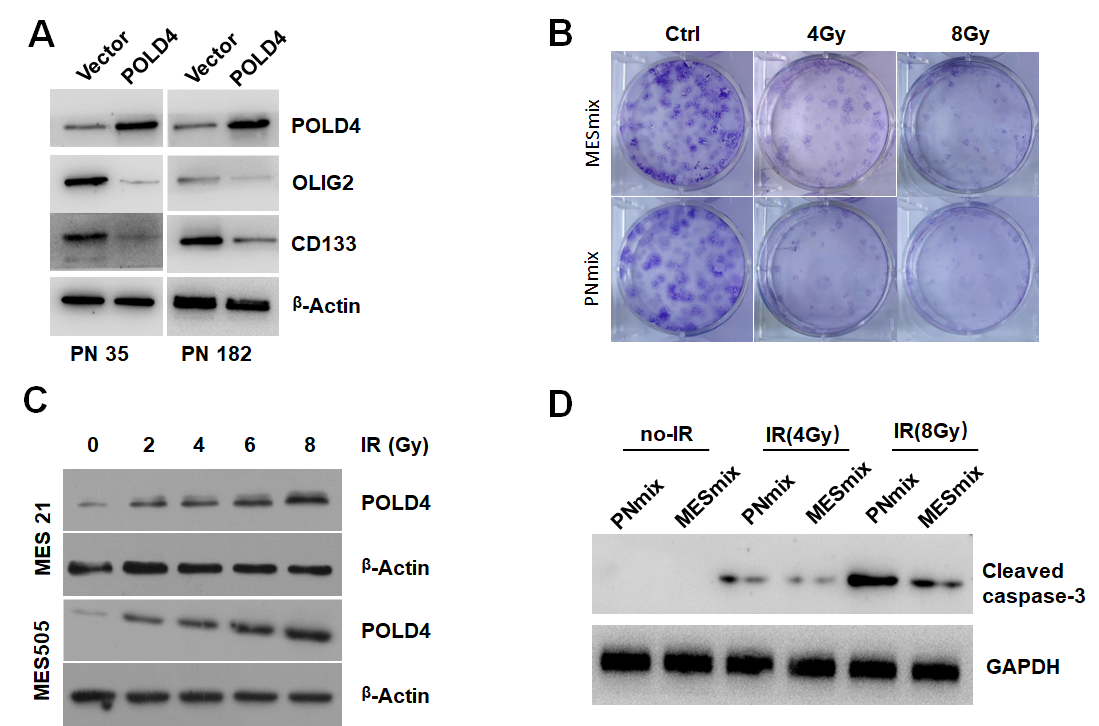
**

Figure S2 (A) IB analysis of POLD4, OLIG2 and CD133 protein levels in PN35 and PN182 overexpressing POLD4 or Vector. (B) The clonogenic images of MES mix (MES 505 and MES 21) cells or PN mix (PN 35 and PN 182) after irradiation with 0-8Gy. (C) IB analysis protein expression of POLD4 in MES 21 and MES 505 under different IR dose treatments. (D) IB analysis protein expression of cleaved caspase-3 in MES mix and PN mix under indicated IR dose treatments.

**Figure S3**

**
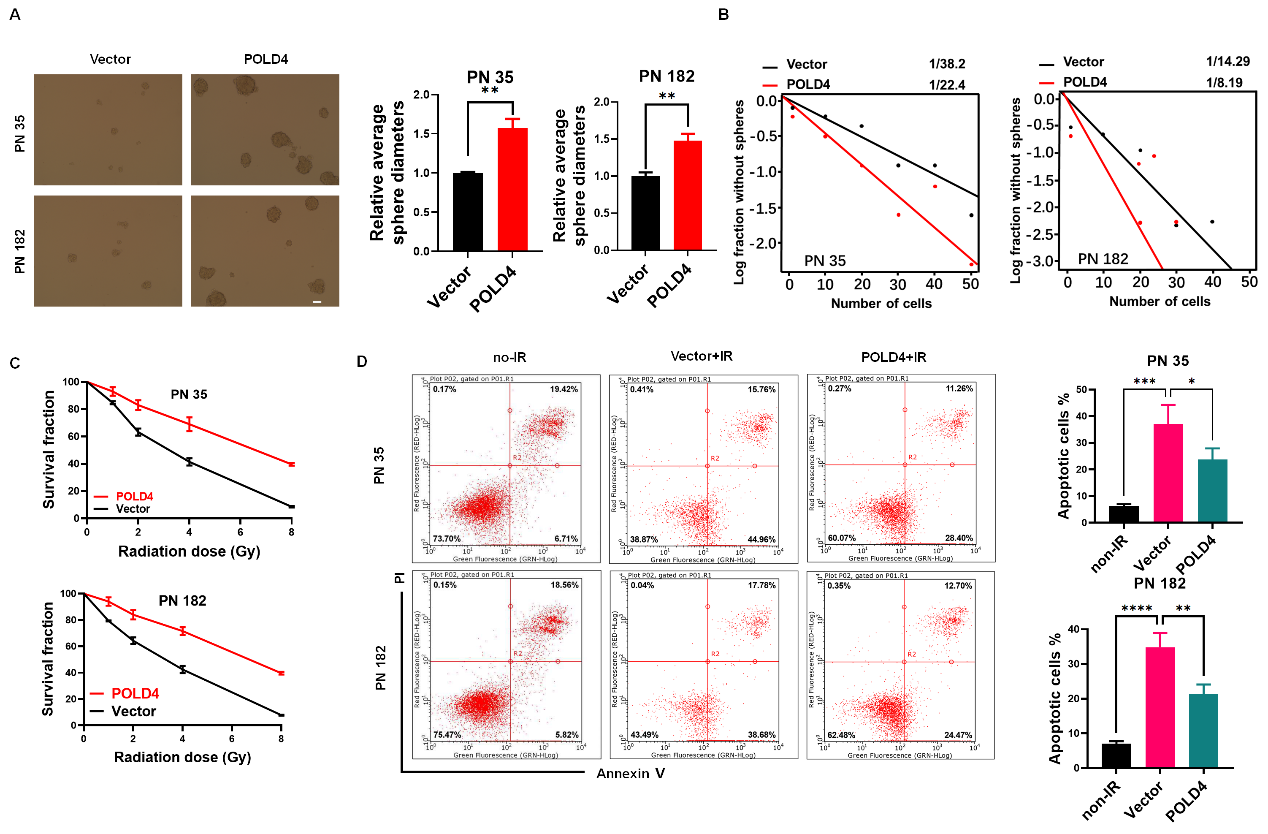
**

Figure S3 (A) Representative images and quantitative analysis for neuro-sphere formation assay of PN 35 and PN 182 transduced with Vector and POLD4 (scale bar, 400 μm). (B) Limiting dilution assays of assay of PN 35 and PN 182 transduced with Vector and POLD4. (C) Radiation survival curves of PN 35 and PN 182 transduced with Vector and POLD4 after irradiation with 0-8Gy. (D) Apoptosis assay was analyzed and quantified by FACScan in PN 35 and PN 182 transduced with Vector and POLD4, in response to IR (6Gy).

**Figure S4**

**
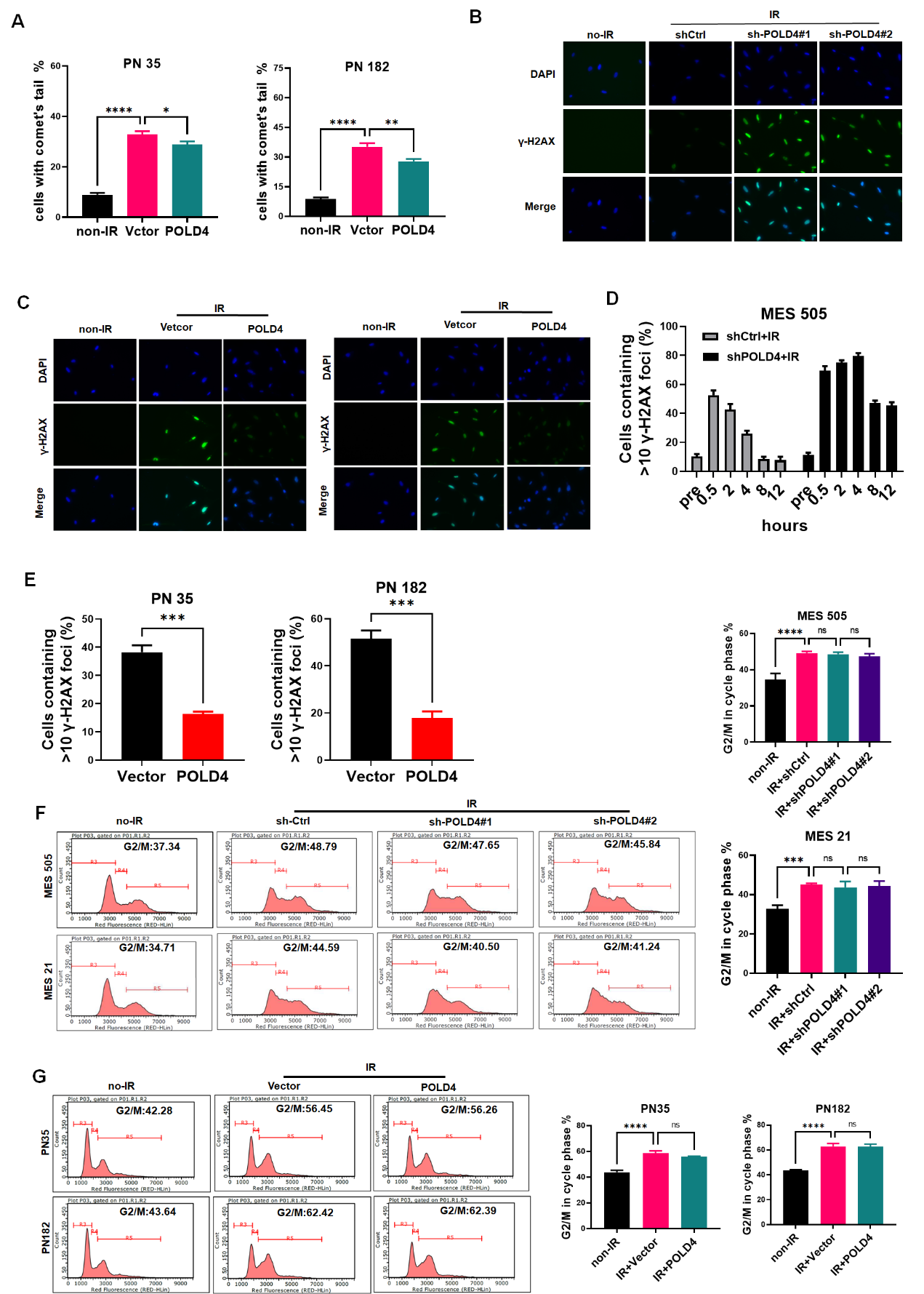
**

Figure S4 (A) Quantification for Comet assays showed different levels of DNA damage in PN 35 and PN 182 expressing Vector and POLD4 with or without irradiated. (B, D) IF analysis (B) and quantification (D) of γ-H2AX foci in MES 505 expressing silencing POLD4 or not, in response to IR. (C, E) Representative images (C) and quantification (E) of γ-H2AX foci in PN 35 and PN 182 expressing Vector and POLD4 with or without irradiated. (F, G) Cell-cycle analysis of MES 21, 505 (F) and PN 35, 182 (G) by FACScan in different treatment groups. The proportions of cells arrested in G2-M phase were quantified.

**Figure S5**

**
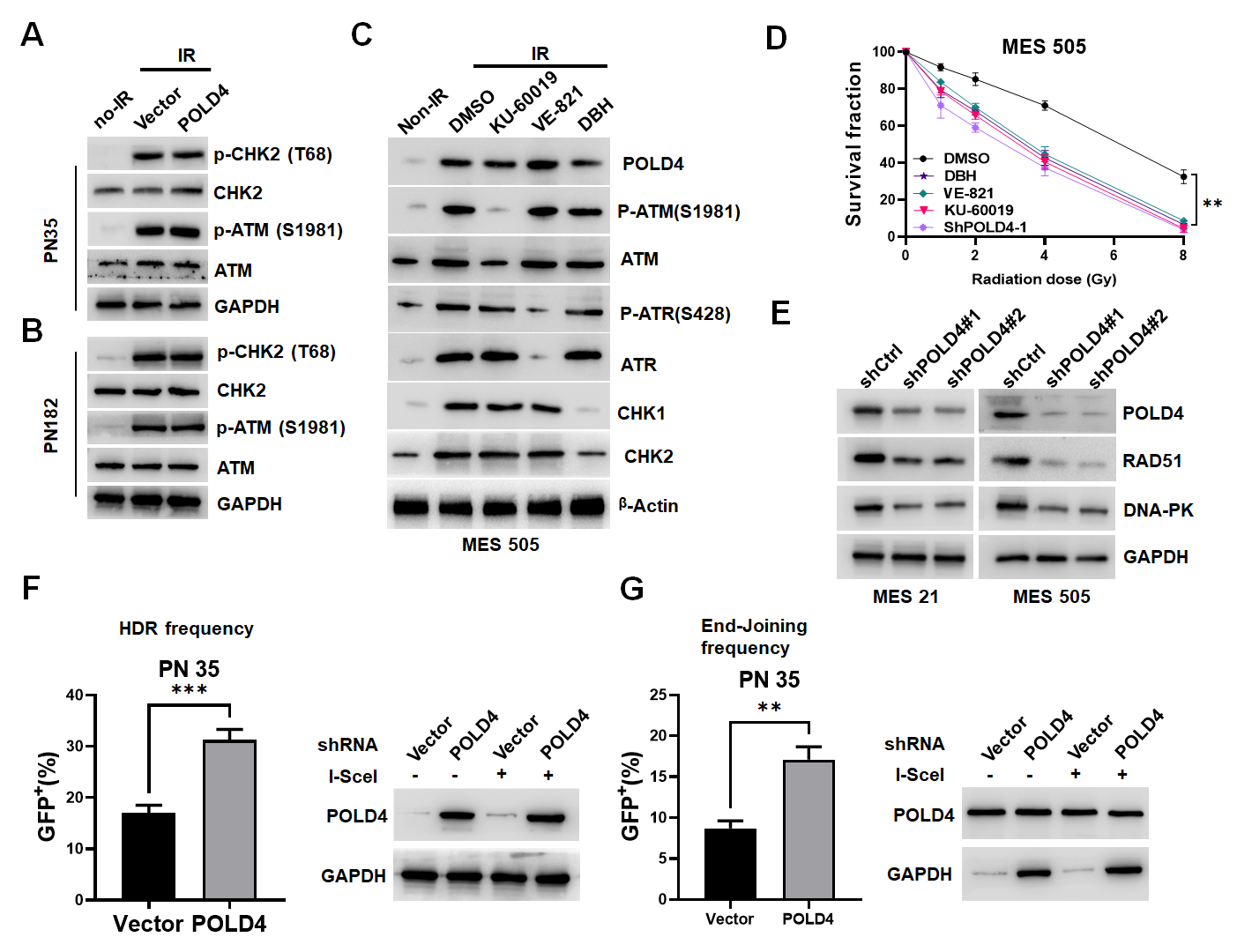
**

Figure S5 (A, B) IB analysis of p-ATM(S1981), p-Chk2 (T68), Chk2, ATM in PN 35 (A) and PN 182 (B) expressing POLD4 or Vector with or without irradiated. (C) IB analysis of POLD4, P-ATM(S1981), P-ATR(S428), ATM, ATR, Chk1, Chk2 in MES 505 treated with DMSO, KU-60019 (selective ATM inhibitor), VE-821 (selective ATR inhibitor), DBH (CHK1/CHK2 inhibitor), in response to IR. (D) Radiation survival curves of MES 505 treated with DMSO, KU-60019, VE-821, DBH, shPOLD4 after irradiation with 0-8Gy. (E) IB assay for DNA-PK,RAD 51 content after POLD4 knockdown (F) PDRGFP stable PN 35 were transfected with POLD4 or Vector. Cells were collected for flow cytometry and IB. (G) PimEJ5GFP stable PN 35 were transfected with POLD4 or Vector. Cells were collected for flow cytometry and IB.

**Figure S6**

**
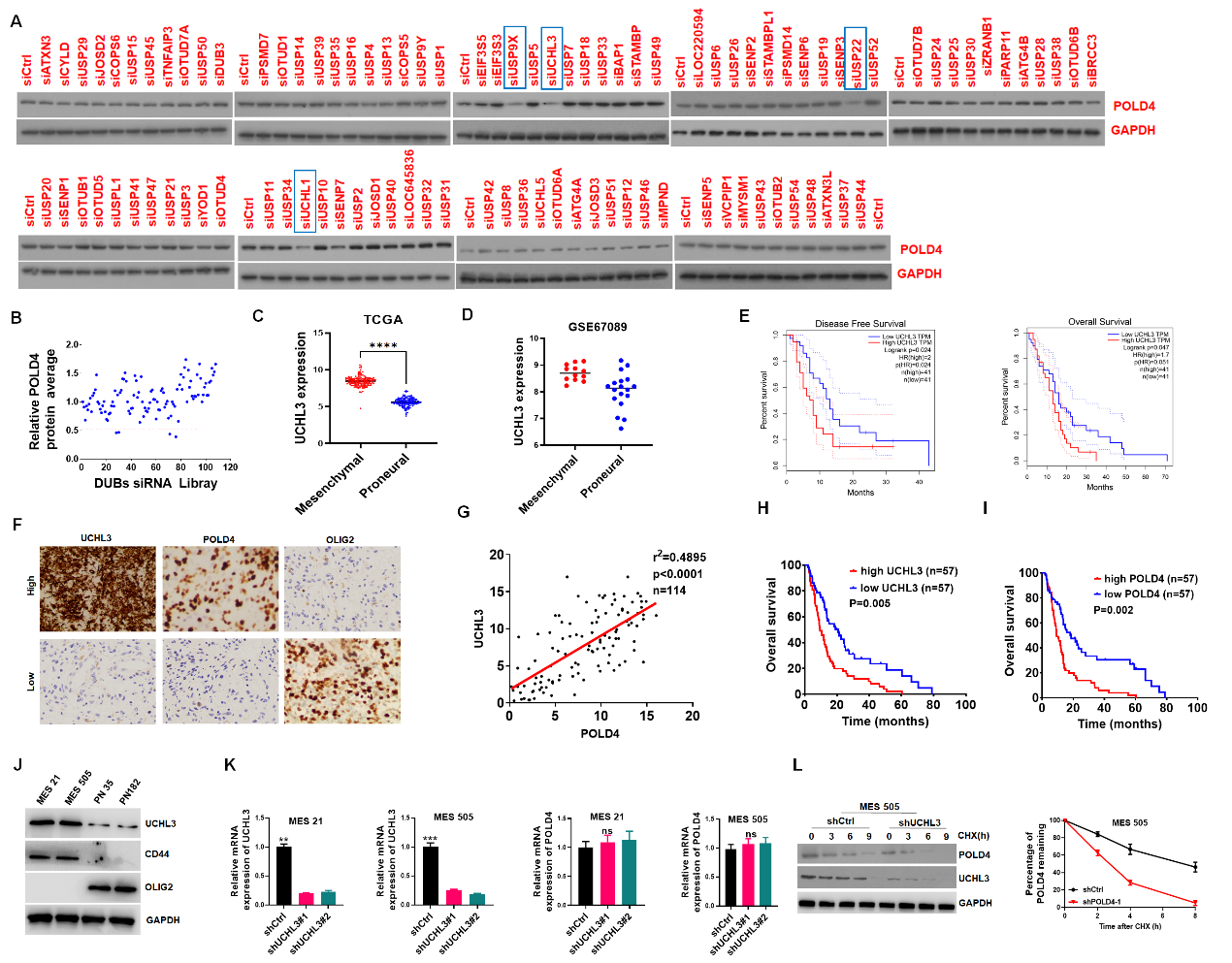
**

Figure S6 (A) IB analysis of POLD4 protein levels with the siGENOME SMARTPool siRNA library targeting 98 annotated human DUBs. (B) quantification of POLD4 levels relative to GAPDH is shown from (A), inclusion criteria for DUBs candidates less than 0.5. (C, D) The expression of UCHL3 mRNA mesenchymal (MES), and proneural (PN) phenotypes in TCGA GBM (C) and GSE67089 (D). (E) Kaplan-Meier overall survival and disease-free survival curves in TCGA GBM patients based on UCHL3 mRNA expression levels. (F) IHC staining of POLD4, UCHL3 and OLIG2 based on tissue sections from 114 formalin-fixed, paraffin-embedded surgical resection specimens, with 2 representative tumors from tissue sections shown. Scale bars: 50μm. (G) Correlation of UCHL3 and POLD4 protein expression levels from 114 GBM specimens was analyzed using Spearman rank correlation analysis. (H, I) Kaplan-Meier curves of disease-free survival (H) and overall survival (I) of 114 patients stratified by low vs. high expression of UCHL3 protein. (J) IB analysis of UCHL3, CD44, and OLIG2 in GCS cells (MES 21, 505; PN 35, 182). (K) qRT-PCR analysis of POLD4 and UCHL3 mRNA expression in MES 21 and 505 GSCs expressing two independent shRNAs targeting UCHL3. (L) MES 505 stably expressing shUCHL3 or shctrl were treated with CHX (0.1 mg/ml) and harvested at the indicated time points for IB analysis of POLD4 and UCHL3 protein levels. For POLD4 decay, quantification of POLD4 levels relative to β-actin is shown.

**Figure S7**

**
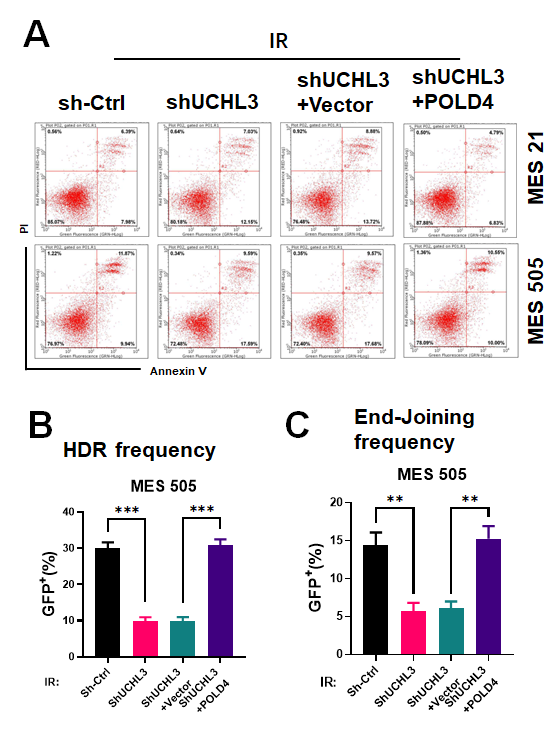
**

Figure S7 (A) Representative images of apoptosis assay in MES 505 and MES 21 by FACScan in different treatment groups. (B) PDRGFP stable MES 505 were transfected with shCtrl or shUCHL3, along with Vector or POLD4, and then exposed to IR(6Gy). Cells were collected for flow cytometry. (C) pimEJ5GFP stable MES 505 were transfected with shCtrl or shUCHL3, along with Vector or POLD4, and then exposed to IR(6Gy). Cells were collected for flow cytometry.

**Figure S8**

**
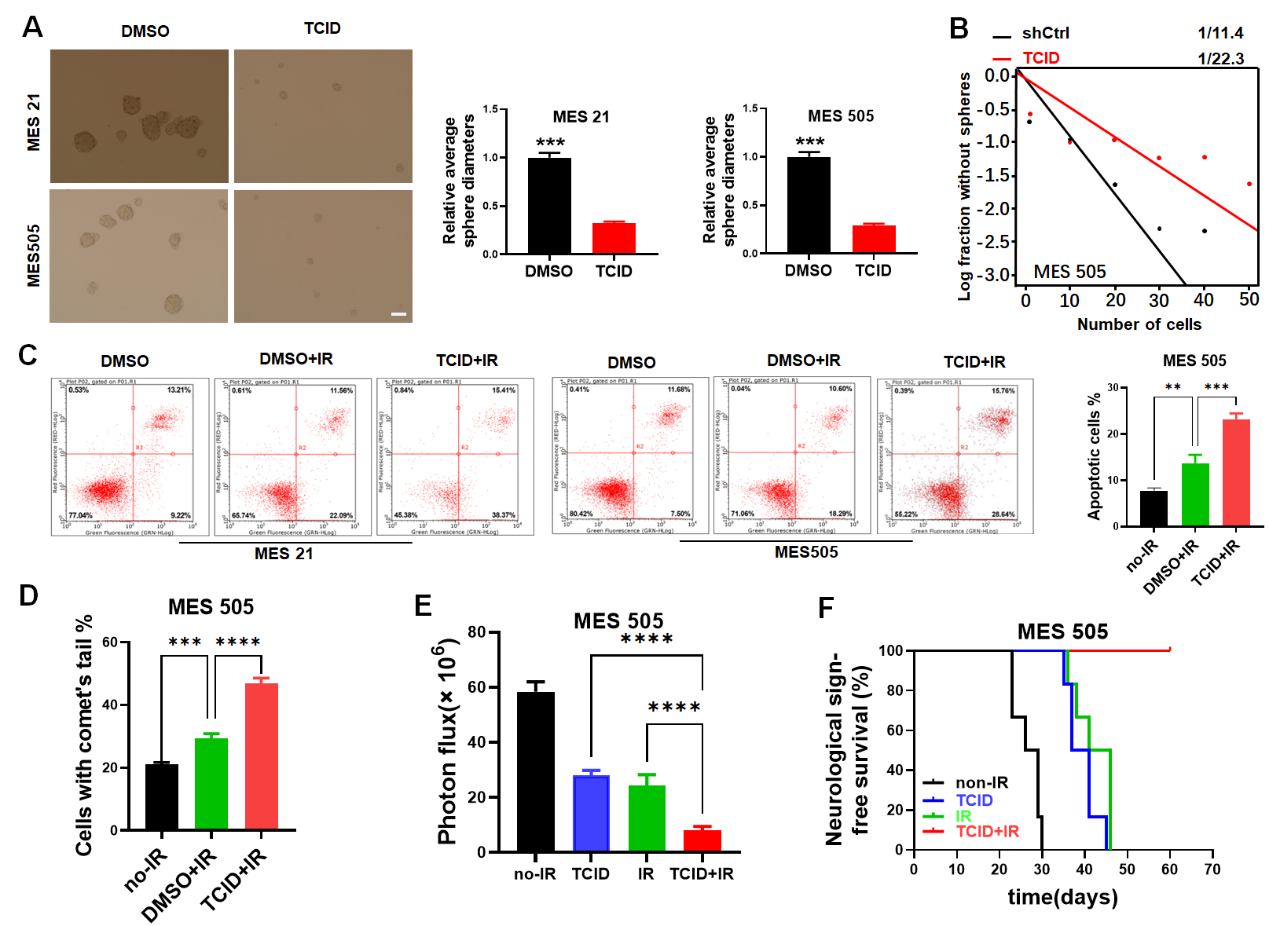
**

Figure S8 (A) Representative images and quantitative analysis for neuro-sphere formation assay of MES 505 and MES 21 with indicated treatment (scale bar, 400 μm). (B) Limiting dilution assays of assay of MES 505 treated with vehicle or TCID. (C) Apoptosis assay was analyzed and quantified by FACScan in MES 505 and MES 21 treated with vehicle or TCID, in response to IR (6Gy). (D) quantification for Comet assays showed different levels of DNA damage in MES 505 following treatment with vehicle or TCID, in response to IR (6Gy). (E)The quantification of tumor photon counts on day 28 after MES 505 intracranial transplantation for mice with indicated treatment. (F) Kaplan–Meier survival curves of mice bearing MES 505-derived xenografts treated with the indicated interventions.

**Table. S1** shRNA and siRNA target sequences used in this study

| Name | Source | Target Sequences |
| --- | --- | --- |
| shUCHL3#1 | Genechem | GCACCAAGTATAGATGAG |
| shUCHL3#2 | Genechem | GTCTTACTTCTCTTTCCTA |
| shUCHL3 3’UTR | Sigma | CTTGTCAATAATGGAAACACC |
| shPOLD4#1 | Sigma | CTGGTGTCGGGCCAAGCAGAT |
| shPOLD4#2 | Sigma | AGTCTCTGGCATCTCTATCCC |
| shPOLD4 3’UTR | Sigma | AGTCAGACATGGACAGTTGAT |
| siUCHL3 | Sigma | Forward (5’-3’): CTGTGTCAATGAGCCCTGAA  Reverse (5’-3’): GCTATGCTGCAGAAAGAGCA |

**Table. S2** Antibodies used in this study

| Antibodies | Source | Identifier |
| --- | --- | --- |
| Anti-UCHL3 | Cell Signaling Technology | 3525S |
| Anti-POLD4 | Proteintech | 26209-1-AP |
| Anti-CD44 | Abcam | ab157107 |
| Anti-CD133 | Abcam | ab284389 |
| Anti-SOX2 | Abcam | ab171380 |
| Anti-OLIG2 | Abcam | ab109186 |
| Anti-Mouse IgG | Abcam | ab190475 |
| Anti-Rabbit IgG | Abcam | ab313802 |
| Anti-TAZ | Abcam | ab307440 |
| Anti-VEGF-A | Abcam | ab46154 |
| Anti-C/EBPβ | Abcam | ab32358 |
| Anti-phospho-STAT3 (Tyr 705) | Cell Signaling Technology | 9131S |
| Anti-STAT3 | Abcam | ab68153 |
| Anti-Ubiquitin | Abcam | ab7254 |
| Anti-HA-Tag | Sigma-Aldrich | sab4300603 |
| Anti-Flag-Tag | Sigma-Aldrich | SAB4301135 |
| Anti-His-Tag | Sigma-Aldrich | SAB1306082 |
| Anti-Cleaved caspase-3 | Abcam | ab2302 |
| Anti-CHK1 | Cell Signaling Technology | 2360S |
| Anti-phospho-CHK2 (T68) | Sigma-Aldrich | SAB4504367 |
| Anti-phospho-ATM (S1981) | Cell Signaling Technology | 4526L |
| Anti-ATM | Cell Signaling Technology | 92356S |
| Anti-phospho-ATR (S428) | Cell Signaling Technology | 2853T-4414288 |
| Anti -ATR | Cell Signaling Technology | 2790S |
| Anti-Myc-Tag | Cell Signaling Technology | 2276S |
| Anti-β-Actin | Cell Signaling Technology | 3700S |
| Anti-GAPDH | Cell Signaling Technology | 97166T |
